# The first 20 months of the COVID-19 pandemic: Mortality, intubation and ICU rates among 104,590 patients hospitalized at 21 United States health systems

**DOI:** 10.1371/journal.pone.0274571

**Published:** 2022-09-28

**Authors:** Michael C. Fiore, Stevens S. Smith, Robert T. Adsit, Daniel M. Bolt, Karen L. Conner, Steven L. Bernstein, Oliver D. Eng, David Lazuk, Alec Gonzalez, Douglas E. Jorenby, Heather D’Angelo, Julie A. Kirsch, Brian Williams, Margaret B. Nolan, Todd Hayes-Birchler, Sean Kent, Hanna Kim, Thomas M. Piasecki, Wendy S. Slutske, Stan Lubanski, Menggang Yu, Youmi Suk, Yuxin Cai, Nitu Kashyap, Jomol P. Mathew, Gabriel McMahan, Betsy Rolland, Hilary A. Tindle, Graham W. Warren, Lawrence C. An, Andrew D. Boyd, Darlene H. Brunzell, Victor Carrillo, Li-Shiun Chen, James M. Davis, Deepika Dilip, Edward F. Ellerbeck, Eduardo Iturrate, Thulasee Jose, Niharika Khanna, Andrea King, Elizabeth Klass, Michael Newman, Kimberly A. Shoenbill, Elisa Tong, Janice Y. Tsoh, Karen M. Wilson, Wendy E. Theobald, Timothy B. Baker

**Affiliations:** 1 Center for Tobacco Research and Intervention, School of Medicine and Public Health, University of Wisconsin School of Medicine and Public Health, Madison, WI, United States of America; 2 Department of Medicine, School of Medicine and Public Health, University of Wisconsin-Madison, Madison, Wisconsin, United States of America; 3 Department of Educational Psychology, University of Wisconsin-Madison, Madison, Wisconsin, United States of America; 4 Department of Emergency Medicine, Geisel School of Medicine at Dartmouth, Lebanon, New Hampshire, United States of America; 5 Institute for Clinical and Translational Research, School of Medicine and Public Health, University of Wisconsin-Madison, Madison, Wisconsin, United States of America; 6 Yale-New Haven Health System, New Haven, Connecticut, United States of America; 7 BlueTree Network, a Tegria Company, Madison, Wisconsin, United States of America; 8 Carbone Cancer Center, University of Wisconsin-Madison, Madison, Wisconsin, United States of America; 9 Department of Family Medicine and Community Health, School of Medicine and Public Health, University of Wisconsin-Madison, Madison, Wisconsin, United States of America; 10 Department of Pediatrics, School of Medicine and Public Health, University of Wisconsin-Madison, Madison, Wisconsin, United States of America; 11 Department of Statistics, University of Wisconsin-Madison, Madison, Wisconsin, United States of America; 12 United States Census Bureau, Washington, D.C., United States of America; 13 Department of Biostatistics & Medical Informatics, University of Wisconsin-Madison, Madison, Wisconsin, United States of America; 14 Department of Human Development, Teachers College, Columbia University, New York, New York, United States of America; 15 Yale School of Medicine, New Haven, Connecticut, United States of America; 16 Department of Population Health Sciences, School of Medicine and Public Health, University of Wisconsin-Madison, Madison, Wisconsin, United States of America; 17 Department of Medicine, Vanderbilt University Medical Center, Nashville, Tennessee, United States of America; 18 Department of Radiation Oncology, Medical University of South Carolina, Charleston, South Carolina, United States of America; 19 Division of General Medicine, Rogel Cancer Center, University of Michigan, Ann Arbor, Michigan, United States of America; 20 Department of Biomedical and Health Information Sciences, College of Applied Health Sciences, University of Illinois at Chicago, Chicago, Illinois, United States of America; 21 Virginia Commonwealth University School of Medicine, Richmond, Virginia, United States of America; 22 Hackensack Meridian Health, Hackensack University Medical Center, Hackensack, New Jersey, United States of America; 23 Washington University in St. Louis School of Medicine, St. Louis, Missouri, USA; 24 Duke Cancer Institute and Duke University Department of Medicine, Durham, North Carolina, United States of America; 25 Memorial Sloan Kettering Cancer Center, New York, New York, United States of America; 26 Department of Population Health, University of Kansas Medical Center, Kansas City, Missouri, United States of America; 27 New York University Langone Health, New York, New York, United States of America; 28 Department of Anesthesiology and Perioperative Medicine, Mayo Clinic, Rochester, Minnesota, United States of America; 29 University of Maryland School of Medicine, Baltimore, Maryland, United States of America; 30 Department of Psychiatry and Behavioral Neuroscience, University of Chicago Comprehensive Cancer Center, Chicago, Illinois, United States of America; 31 Department of Preventive Medicine, Northwestern University Feinberg School of Medicine, Chicago, Illinois, United States of America; 32 University of Utah, Salt Lake City, Utah, United States of America; 33 Department of Family Medicine and Lineberger Comprehensive Cancer Center, University of North Carolina School of Medicine, Chapel Hill, North Carolina, United States of America; 34 University of California Davis, Davis, California, United States of America; 35 Department of Psychiatry and Behavioral Sciences, Hellen Diller Family Comprehensive Cancer Center, University of California San Francisco, San Francisco, California, United States of America; 36 Department of Pediatrics, University of Rochester School of Medicine, Rochester, New York, United States of America; Samsung Medical Center, REPUBLIC OF KOREA

## Abstract

**Main objective:**

There is limited information on how patient outcomes have changed during the COVID-19 pandemic. This study characterizes changes in mortality, intubation, and ICU admission rates during the first 20 months of the pandemic.

**Study design and methods:**

University of Wisconsin researchers collected and harmonized electronic health record data from 1.1 million COVID-19 patients across 21 United States health systems from February 2020 through September 2021. The analysis comprised data from 104,590 adult hospitalized COVID-19 patients. Inclusion criteria for the analysis were: (1) age 18 years or older; (2) COVID-19 ICD-10 diagnosis during hospitalization and/or a positive COVID-19 PCR test in a 14-day window (+/- 7 days of hospital admission); and (3) health system contact prior to COVID-19 hospitalization. Outcomes assessed were: (1) mortality (primary), (2) endotracheal intubation, and (3) ICU admission.

**Results and significance:**

The 104,590 hospitalized participants had a mean age of 61.7 years and were 50.4% female, 24% Black, and 56.8% White. Overall risk-standardized mortality (adjusted for age, sex, race, ethnicity, body mass index, insurance status and medical comorbidities) declined from 16% of hospitalized COVID-19 patients (95% CI: 16% to 17%) early in the pandemic (February-April 2020) to 9% (CI: 9% to 10%) later (July-September 2021). Among subpopulations, males (vs. females), those on Medicare (vs. those on commercial insurance), the severely obese (vs. normal weight), and those aged 60 and older (vs. younger individuals) had especially high mortality rates both early and late in the pandemic. ICU admission and intubation rates also declined across these 20 months.

**Conclusions:**

Mortality, intubation, and ICU admission rates improved markedly over the first 20 months of the pandemic among adult hospitalized COVID-19 patients although gains varied by subpopulation. These data provide important information on the course of COVID-19 and identify hospitalized patient groups at heightened risk for negative outcomes.

**Trial registration:**

ClinicalTrials.gov Identifier: NCT04506528 (https://clinicaltrials.gov/ct2/show/NCT04506528).

## Introduction

COVID-19 (“COVID”) has disrupted virtually every aspect of society, infecting about 80 million individuals in the U.S. and causing almost one million COVID deaths through March 2022 [1]. It is important to track COVID outcomes over time, particularly among hospitalized COVID patients who are at heightened risk of severe outcomes. Such examination can inform clinical care, guide public health actions, influence policy, and identify vulnerable populations.

Cohort studies have shown meaningful changes in mortality throughout the COVID pandemic, although most reported outcomes are from early in the pandemic [2–6], often with modest sized samples. Such studies found decreasing mortality rates amongst COVID cases over time [2, 3, 5, 7, 8], and increased mortality when hospital admission rates for COVID were high [5, 9]. The National COVID Cohort Collaborative (N3C) retrospective cohort study [10] examined predictors of COVID mortality for 32,472 U.S. adults who were hospitalized with COVID between January 1, 2020, and December 7, 2020. Electronic health record (EHR) data showed that COVID related mortality decreased throughout the study. However, data on medical comorbidities were present in only 49% of N3C patients.

The present retrospective cohort study reports changes in COVID-associated mortality, intubation, and ICU admission rates among 104,590 patients hospitalized with COVID at 21 U.S. health systems from February 1, 2020, to September 30, 2021.

## Methods

### Study design

The COVID EHR Cohort at the University of Wisconsin (CEC-UW) is a retrospective cohort study. Presented results include data from February 1, 2020, to September 30, 2021. Health systems from across the U.S. were invited to participate and 21 joined the cohort (S1 Fig in S1 File) and transferred data regularly to the CEC-UW Coordinating Center in Madison, Wisconsin. Each data transfer included data dating back to February 1, 2020.

### Ethics statement

The CEC-UW study was initially approved in May 2020 by the University of Wisconsin-Madison Health Sciences Minimal Risk Institutional Review Board (MR-IRB) with approval for the collection of de-identified EHR data from the 21 health systems. The MR-IRB also determined that the study met criteria for a human subjects research exemption and qualified for a waiver of informed consent under the Federal Common Rule. All participating health systems provided written notice of either their own institution’s IRB approval or determination of exemption status before sharing EHR data. In February 2021, the MR-IRB approved a change of protocol for a Limited Data Set, allowing the collection of additional information (e.g., death dates, five-digit zip codes) but excluding direct patient identifiers. Each patient in the data set from each health system was assigned an enduring cryptographically processed Patient ID based on the SHA256 algorithm, which yielded a 64-character unique and private hash-based message authentication code (HMAC). Study reporting follows STROBE guidelines (S1 Method in S1 File).

### Data collection

#### Extraction, harmonization, and secure transfer of EHR data

EHR data extraction code was created by programmers at UW School of Medicine and Public Health (Madison, WI), Yale New Haven Health (New Haven, CT), and Bluetree Network, Inc. [11]. Data elements were extracted for patient sociodemographic variables, general health information, clinical encounter data, pre-COVID and post-COVID ICD-10 diagnoses, laboratory test results, and medication information (S3 Method in S1 File).

The extraction code was customized at each health system to map to their EHR data to yield relatively uniform data sets. Additional data harmonization and quality assurance was done by CEC-UW staff (S3 Method in S1 File). Secure transfer of data from each of the 21 health systems was accomplished via the transfer of data files to a secure SFTP (secure shell [SSH] File Transfer Protocol) portal located at the UW-Madison CEC-UW Coordinating Center.

#### Extracted data categories

Each health system transferred five source data files (S3 Method in S1 File) with patient- and encounter-level information on: 1) sociodemographic and health characteristics; 2) pre- and post-COVID ICD-10 diagnoses; 3) clinical encounter data including treatment site (e.g., inpatient, outpatient), encounter-based ICD-10 diagnoses, mortality, ICU admission, intubation, and other clinical data; 4) selected laboratory test results linked to encounters; and 5) selected medications linked to encounters. Health systems provided data only for closed clinical encounters (i.e., completed). For closed inpatient encounters, the patient must have been discharged or died during the hospitalization. Data on outcomes or treatment at nonparticipating health systems were not captured.

### Analysis sample

**Fig 1:**
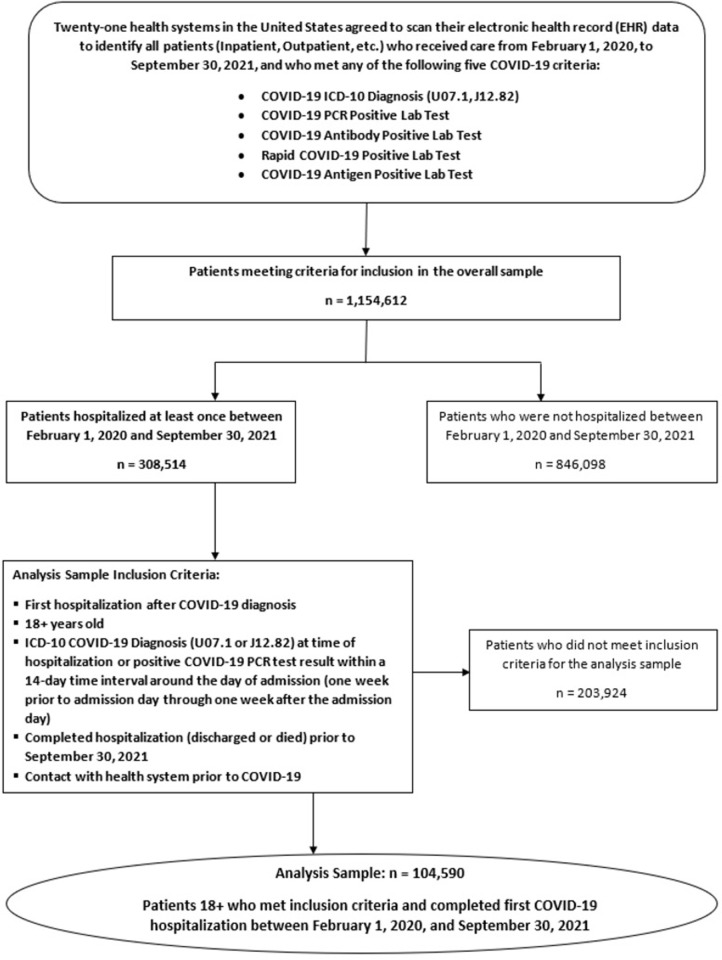
CEC–UW: Cohort criteria for inclusion in the analyzed sample of 104,590 hospitalized COVID–19 patients.

The analysis sample comprised 104,590 adult patients hospitalized with COVID (Fig 1 and S2 Method in S1 File). Analysis sample inclusion criteria included: 1) ≥18 years old; 2) the inpatient encounter was the first COVID hospitalization with duration ≥ 24 hours (or, if < 24 hours, admission to ICU or death during the hospitalization); 3) COVID ICD-10 diagnosis (U07.1 or J12.82) during the hospitalization; 4) positive COVID PCR test result in a 14-day window (+/- 7 days centered at the admission date); and 5) prior contact with the health system to permit extraction of pre-COVID ICD-10 diagnoses to calculate the Elixhauser Comorbidity Score [12] (S5 Method in S1 File). Overall, 73.0% (n = 76,303) of the sample had both a positive PCR test result and a COVID ICD-10 diagnosis, 6.8% (n = 7,118) had only a positive PCR test, and 20.2% (n = 21,169) had only a COVID ICD-10 diagnosis at the time of hospitalization.

### Primary and secondary outcomes

The primary outcome was in-hospital mortality during the index COVID hospitalization documented via EHR. Secondary outcomes included: (1) endotracheal intubation and (2) ICU admission during hospitalization. All outcomes were binary.

### Non-outcome variables

Patient-level variables include age (at time of entry into the cohort), sex, race, ethnicity, body mass index (BMI), insurance status, Elixhauser Comorbidity Score, and vaccination status (yes/no). Patients aged ≥ 90 years were coded as 90 at the time of data extraction. For certain analyses, age was categorized as: 18–59 years, 60–70 years, and over 70 years (cut-points suggested by class probability trees). See Table 1 for race, ethnicity, BMI categories, and insurance status categories. Race and ethnicity categories were based on definitions used by the National Institutes of Health [13]. The Elixhauser Comorbidity Score was calculated [12] using van Walraven weights (S5 Method in S1 File) based on ICD-10 diagnoses (present vs. absent) determined via a 5-year look back pre-COVID.

**Table 1:** Descriptive statistics for 104,590 hospitalized COVID–19 patients from February 2020 to September 2021.

Variable	Mean (Standard Deviation [SD] or Frequency (Percentage)(N = 104,590 Adult COVID Patients)
Age in years	61.7 (SD = 18.0)
Age Groups	Under 60 Years: 43,205 (41.3%)
	Age 60–70 Years: 24,440 (23.4%)
	Over 70 Years: 36,945 (35.3%)
Sex	Female: 52,701 (50.4%)
	Male: 51,887 (49.6%)
	Other: 2 (<0.01%)
Race	American Indian/Alaska Native: 389 (0.4%)
	Asian: 3,047 (2.9%)
	Black or African American: 25,073 (24.0%)
	Native Hawaiian or Other Pacific Islander: 484 (0.5%)
	White: 59,362 (56.8%)
	Other Race: 14,116 (13.5%)
	More Than One Race: 366 (0.3%)
	Missing: 1,753 (1.7%)
Ethnicity	Not Hispanic or Latino: 84,827 (81.1%)
	Hispanic or Latino: 16,661 (15.9%)
	Missing: 3,102 (3.0%)
Body Mass Index	Underweight: 3,042 (2.9%)
	Healthy Weight: 23,483 (22.5%)
	Overweight: 29,940 (28.6%)
	Obese: 35,095 (33.6%)
	Severely Obese: 11,997 (11.5%)
	Missing: 1,033 (1.0%)
Insurance Status	Medicare: 55,427 (53.0%)
	Medicaid: 12,177 (11.6%)
	Commercial: 27,921 (26.7%)
	Uninsured: 1,967 (1.9%)
	Other/Missing: 7,098 (6.8%)

### Statistical analysis

#### Statistical analysis plan

A detailed statistical analysis plan [14] was prepared by CEC-UW scientists and reviewed by a Data Analytic Consulting Committee comprising NCI methodologists.

#### Descriptive statistics and missingness

Descriptive statistics for the analysis sample characteristics and selected outcome analyses were computed using SAS version 9.4 (SAS Institute Inc). There were no missing data for the primary or secondary outcomes. Missing data for covariates are reported in Table 1.

#### Calculation of health system risk standardized mortality rates for health system/month

**Fig 2:**
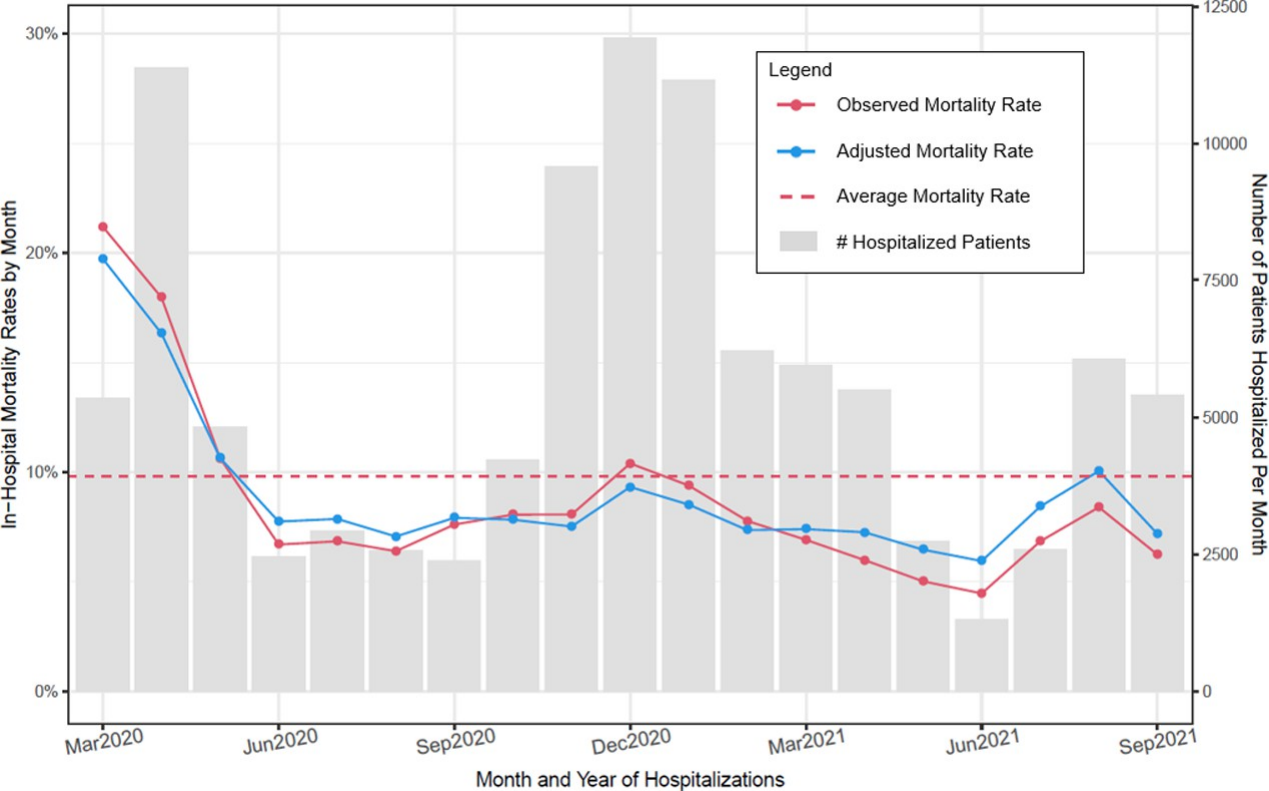
Observed and adjusted in–hospital mortality rates and number of patients hospitalized per month across 21 health systems (March 2020 through September 2021).

Risk standardized mortality rates (RSMRs) adjusting for health systems and months followed a strategy presented by Silber and colleagues [15–18] (S6 Method in S1 File). Covariates included age, sex, race, ethnicity, BMI, insurance status, Elixhauser score, and health system to control for such factors in comparing outcomes across health systems within months (S6 Fig in S1 File) and across months when pooling health systems (Fig 2 and S11, S12 Figs in S1 File). The same generalized linear mixed modeling (GLMM) strategy used for calculating RSMRs for health system was also used in calculating RSMRs for each month of the study period by interchanging health system random effects with month random effects. Thus, in some figures (Fig 2 and S11, S12 Figs in S1 File), the results of adjustments account solely for changes in the aggregate distributions of patient characteristics by month. S6 Fig in S1 File plots the health system RSMRs by month, adjusting for variability in patient characteristics across health system within month. Missingness occurred in only categorical covariates and was treated as its own category; its disproportionately strong association with negative outcomes argued against imputation.

#### Changes in outcomes

To evaluate changes over time in mortality, we compared rates for the first three months of the study (February 2020 to April 2020, Period 1) to rates for the last three months of the study (July 2021 to September 2021, Period 2). This *post hoc* approach captured change from the peak of mortality early in the pandemic to rates at the end of the 20-month study period (as per Asch et al [3]).

Changes in rates of mortality (Period 1 versus Period 2) were computed along with logistic regression-based odds ratios (ORs), 95% confidence intervals (CIs), and p-values. Changes in overall mortality were also analyzed for the patient-level covariates: age, sex, race, ethnicity, BMI, insurance status, and Elixhauser score; vaccination history was also used as a covariate in focused analyses. Effects are presented with and without covariate adjustment. The latter has clinical relevance since it reflects associations with person characteristics as they present in healthcare (where such characteristics co-occur with other risk factors). Alpha was set at .05 (two-tailed test); corrections for multiple tests were not made.

## Results

Fig 1 depicts the full CEC-UW cohort (N = 1,154,612) drawn from the 21 health systems from February 1, 2020, to September 30, 2021, and shows the 104,590 hospitalized COVID patients meeting the analysis sample inclusion criteria.

### Characteristics of the participating health systems

The number of analysis sample patients from each of the 21 participating health systems ranged from 386 to 15,584 (mean = 4,980 patients, SD = 4,104; median = 3,100).

### Characteristics of analysis sample patients

Table 1 provides descriptive statistics for patient characteristics of the analysis sample. Month-by-month distributions of patient age (S2 Fig in S1 File), Elixhauser scores (S3 Fig in S1 File), and length of hospital stay in non-deceased patients (S4 Fig in S1 File) are provided in the supporting information.

### Hospital admission rates over time

Fig 2 presents month-by-month hospital admission rates for COVID patients (as gray bars) over the study period collapsing across the 21 health systems. This figure reveals several peaks of admissions across the 20-month period. Rates for February 2020 were omitted from figures due to the small N (total N = 242).

### Unadjusted and risk standardized mortality rates (RSMRs)

Fig 2 shows both the unadjusted (i.e., observed) rates and RSMRs per month collapsing across the 21 health systems. Fig 2 shows that the highest mortality rates occurred early in the pandemic in March and April 2020. However, there were modest increases in mortality rates during two later peaks in hospital admissions (December 2020 to January 2021 and July to August 2021). The temporal patterns in mortality over time were very similar in RSMR and unadjusted analyses (Fig 2). Across the health systems, mortality decreased from the first three months of the pandemic (February-April 2020) to the last three months of the study period (July 2021 to September 2021) from 18.6% (95% CI: 18% to 19%) to 7.3% (95% CI: 7% to 7.8%) (unadjusted) and from 16.4% (95% CI = 16% to 17%) to 9% (95% CI: 9% to 10%) (adjusted). However, Fig 2 shows that mortality rates appeared to plateau after the first several months of the pandemic and gains were slight after.

There was substantial variability in unadjusted mortality across the different health systems (S5 Fig in S1 File), and a general trend of decreasing mortality from early in the pandemic with rates plateauing thereafter. RSMR adjustment across health systems substantially decreased health system variability (S6 Fig in S1 File).

### Mortality rates as a function of each of six patient-level variables

**Fig 3:**
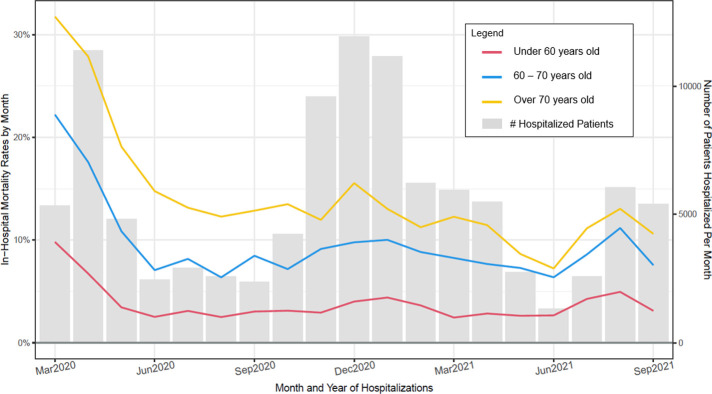
Observed in–hospital mortality rates (Deaths/hospitalizations) and number of patients hospitalized per month for patient age groups (March 2020 through September 2021).

**Fig 4:**
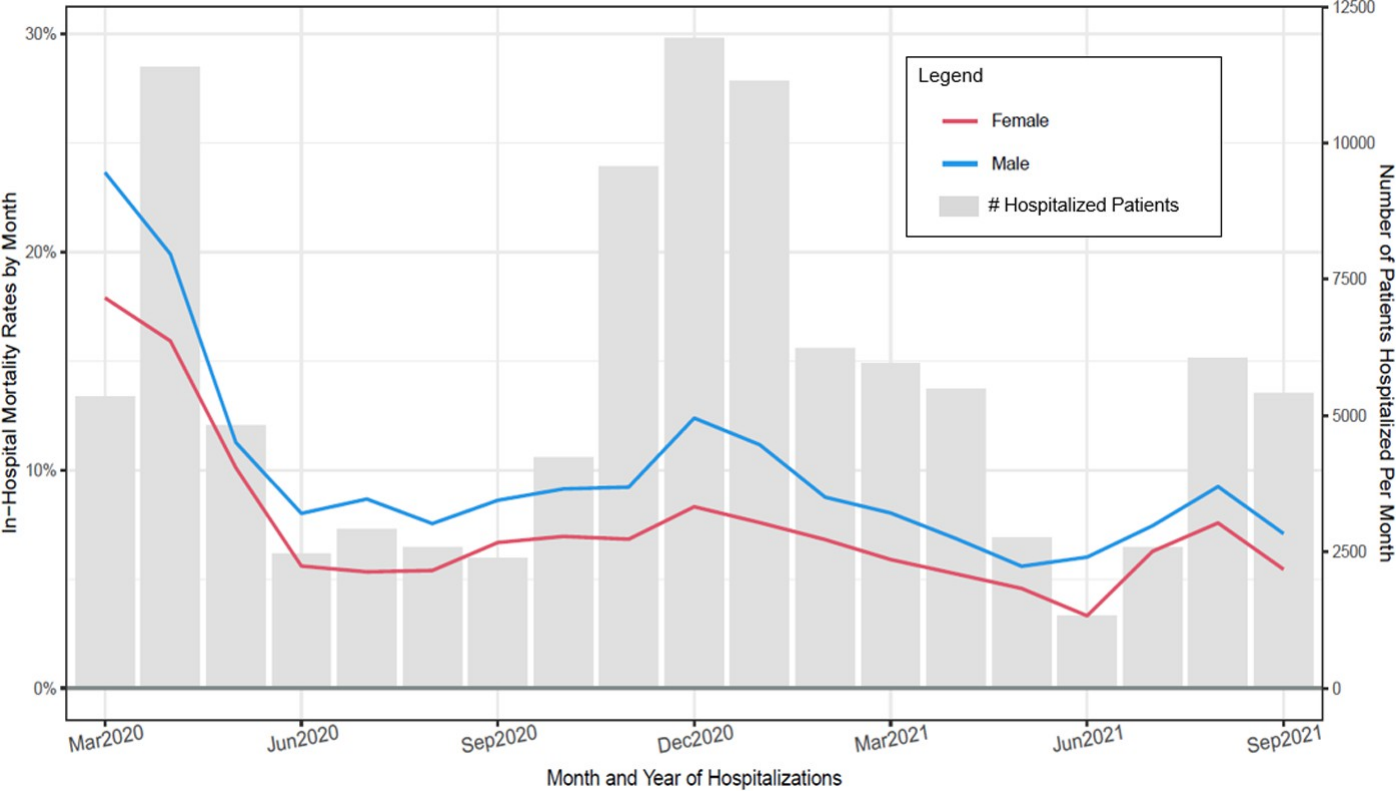
Observed in–hospital mortality rates (Deaths/hospitalizations) and number of patients hospitalized per month for patient sex groups (March 2020 through September 2021).

**Fig 5:**
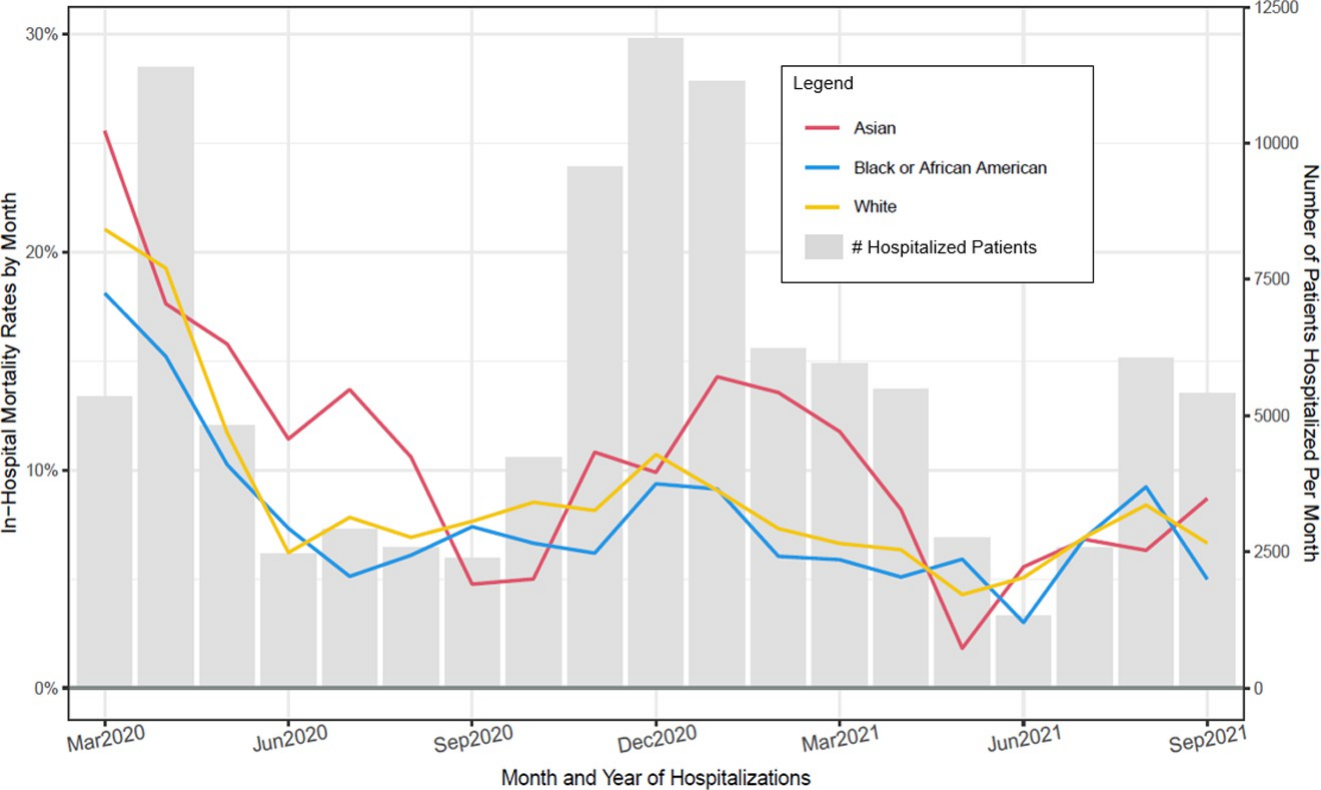
Observed in–hospital mortality rates (Deaths/hospitalizations) and number of patients hospitalized per month for patient race groups (March 2020 through September 2021).

**Fig 6:**
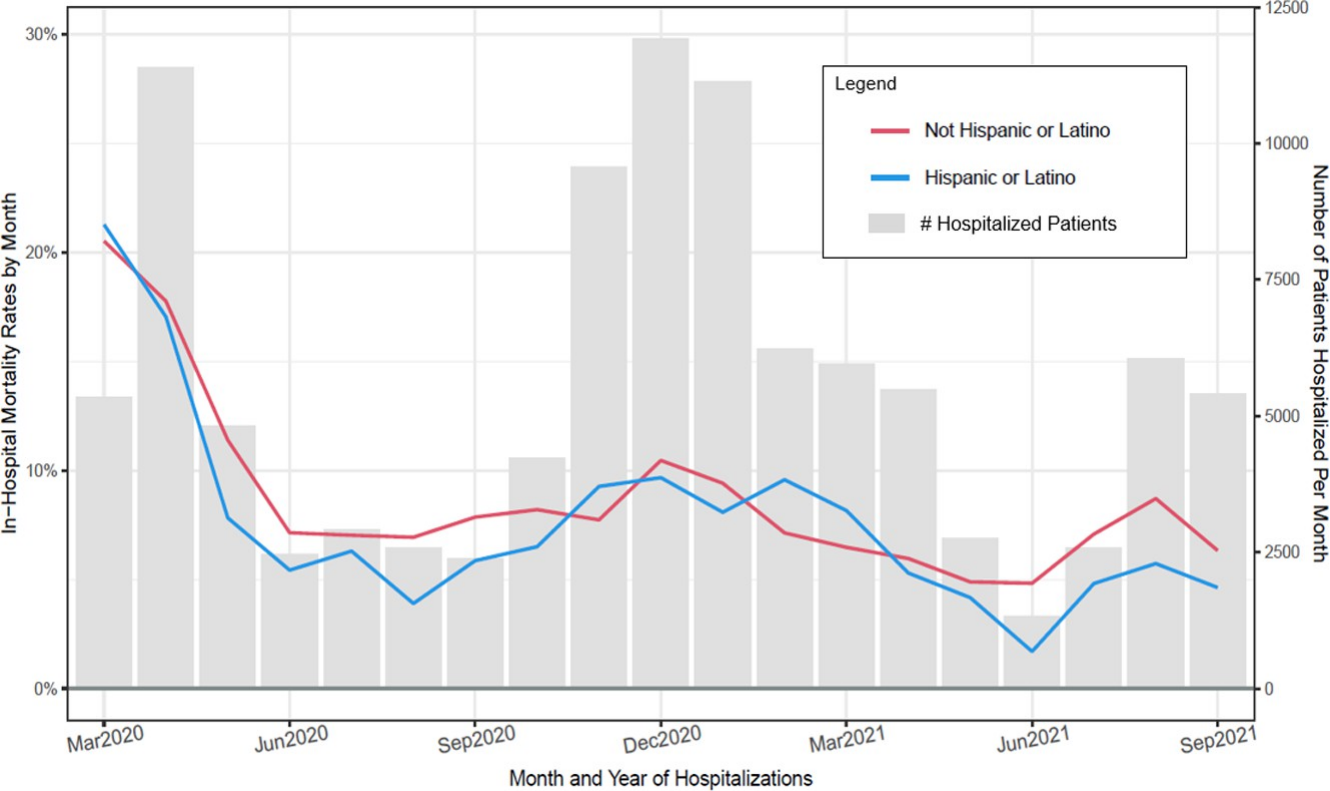
Observed in–hospital mortality rates (Deaths/hospitalizations) and number of patients hospitalized per month for patient ethnicity subpopulations (March 2020 through September 2021).

**Fig 7:**
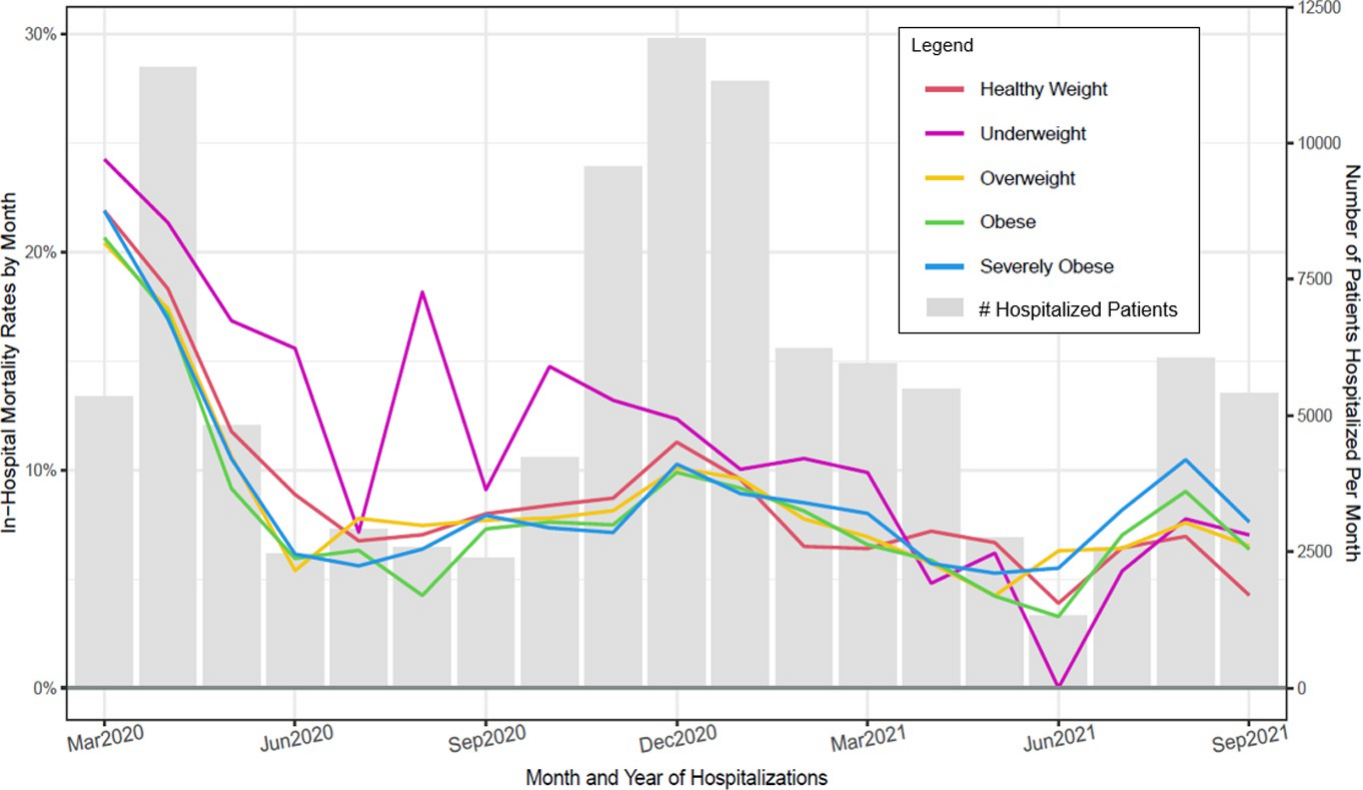
Observed in–hospital mortality rates (Deaths/hospitalizations) and number of patients hospitalized per month for patient BMI groups (March 2020 through September 2021).

**Fig 8:**
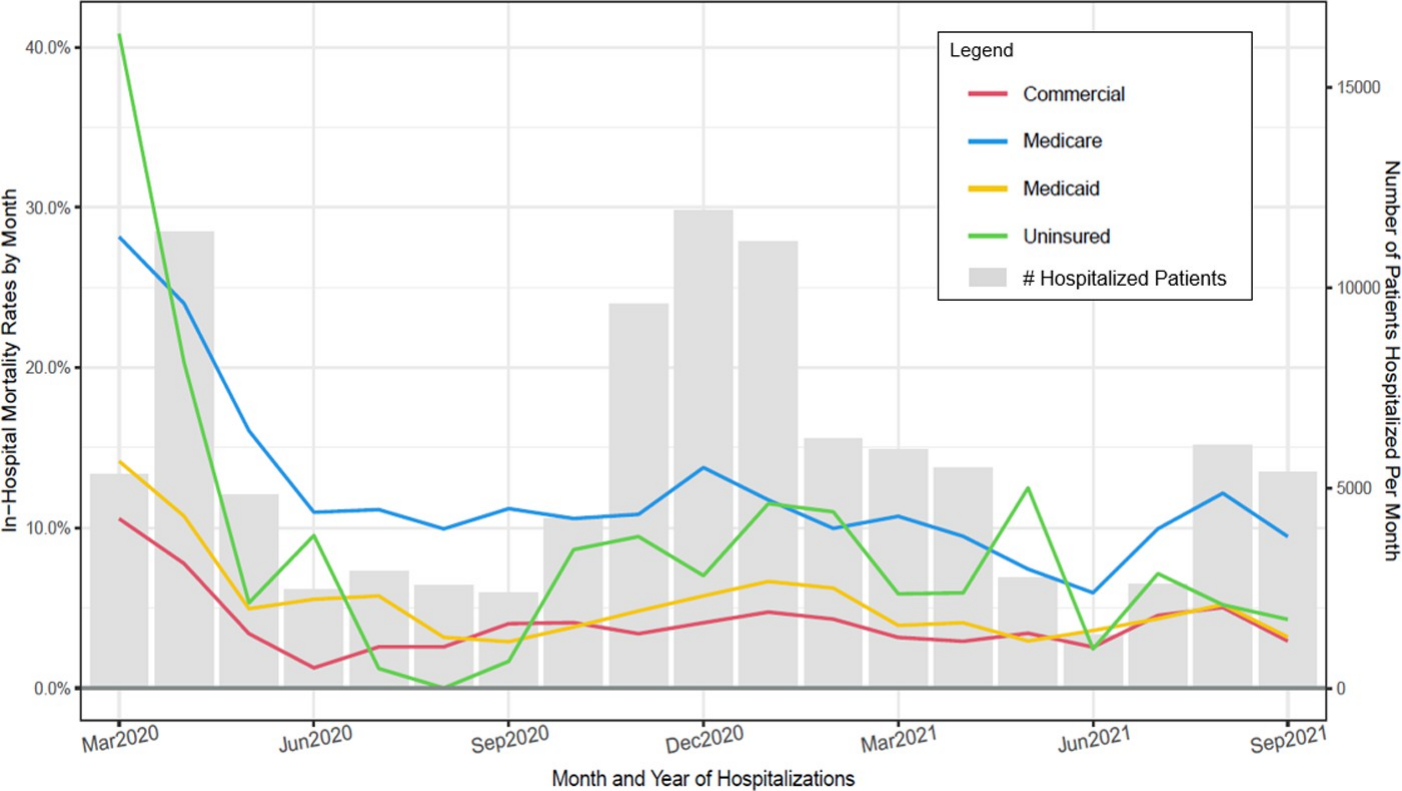
Observed in–hospital mortality rates (Deaths/hospitalizations) and number of patients hospitalized per month for patient insurance status groups (March 2020 through September 2021).

Figs 3–8 show unadjusted monthly mortality rates as a function of each of the six patient-level variables. Values for patient-level subpopulations of very small size and values for missing data are not shown in Figs 3–8 (All subpopulations are shown in S7-S10 Figs in S1 File).

### Intubation and ICU admission rates over time

S11 Fig in S1 File shows monthly intubation rates and S12 Fig in S1 File shows monthly ICU admission rates for March 2020 to September 2021 across the 21 health systems. S11 Fig in S1 File shows that intubation rates decreased early in the 20-month period and then largely plateaued. S12 Fig in S1 File shows that ICU admission rates varied substantially over time, increasing when hospital admission rates declined. Thus, the pattern observed with regard to intubation paralleled that observed with mortality, plateauing after the initial months of the pandemic. ICU admission may more closely reflect hospital policies and resources and admission surge rates leading to greater variation over time.

### Change in mortality as a function of patient characteristics

Table 2 displays logistic regression results for the predictive effects of patient variables regarding mortality and mortality change: 1) unadjusted and adjusted effects of each patient variable (e.g., each race comparison category against its reference category—White) *within* the first three-month period (Period 1: February-April 2020) and *within* the last three-month period (Period 2: July-September 2021); 2) observed change within each patent variable category *across* the two time periods; and 3) differential effects of patient variables across time and in relation to mortality change observed in the reference condition. The adjusted effects reported for each patient variable category include the Elixhauser comorbidity index and the remaining tabled patient variables as covariates.

**Table 2:** Mortality rates for hospitalized COVID–19 patients during the first three and final three months of data collection.

Patient Variable	Period 1: February-April, 2020			Period 2: July-Sept, 2021			Change	
	Mortality Rate Deaths/Total Hosp.	OR (95% CI)	*p*	Mortality Rate Deaths/Total Hosp.	OR (95% CI)	*p*	OR (95% CI)	*p*
	N (%)			N (%)				
**Sex**								
Female (**REF)**	1296/7844 (16.5)	1.00		467/7176 (6.5)	1.000		0.35 (0.32, 0.39)	< .001
Male	1887/8909 (21.2)	1.36 (1.26, 1.47)	< .001	556/6868 (8.1)	1.27 (1.11, 1.44)	< .001	0.32 (0.30, 0.36)	< .001
*Adjusted*		*1*.*53 (1*.*40*, *1*.*66)*	*<* .*001*		*1*.*32 (1*.*16*, *1*.*51)*	*<* .*001*	*0*.*87 (0*.*74*, *1*.*01)*	.*070*
**Race**								
White (**REF**)	1509/7599 (19.9)	1.00		670/8995 (7.4)	1.000		0.33 (0.30, 0.36)	< .001
American Indian	10/41 (24.4)	1.30 (0.64, 2.66)	.470	8/57 (14.0)	2.03 (0.96, 4.30)	.065	0.51 (0.18, 1.42)	.196
*Adjusted*		*1*.*88 (0*.*88*, *4*.*02)*	.*105*		*2*.*44 (1*.*12*, *5*.*30)*	.*025*	*1*.*30 (0*.*44*, *3*.*85)*	.*639*
Asian	137/680 (20.1)	1.02 (0.84, 1.24)	.856	15/208 (7.2)	0.97 (0.57, 1.64)	.898	0.31 (0.18, 0.54)	< .001
*Adjusted*		*1*.*37 (1*.*12*, *1*.*69)*	.*003*		*1*.*36 (0*.*79*, *2*.*34)*	.*272*	*0*.*99 (0*.*55*, *1*.*77)*	.*967*
Black	686/4256 (16.1)	0.78 (0.70, 0.86)	< .001	241/3352 (7.2)	0.96 (0.83, 1.12)	.625	0.40 (0.35, 0.47)	< .001
*Adjusted*		*0*.*93 (0*.*84*, *1*.*03)*	.*175*		*1*.*16 (0*.*99*, *1*.*37)*	.*067*	*1*.*25 (1*.*03*, *1*.*52)*	.*023*
Pacific Islander	13/118 (11.0)	0.50 (0.28, 0.89)	.019	3/39 (7.7)	1.04 (0.32, 3.37)	.954	0.67 (0.18, 2.50)	.554
*Adjusted*		*0*.*88 (0*.*48*, *1*.*60)*	.*674*		*1*.*23 (0*.*37*, *4*.*10)*	.*733*	*1*.*40 (0*.*37*, *5*.*36)*	.*623*
Other/Not Spec.	753/3654 (20.6)	1.05 (0.95, 1.16)	.353	63/1118 (5.6)	0.74 (0.57, 0.97)	.028	0.23 (0.17, 0.30)	< .001
*Adjusted*		*1*.*40 (1*.*22*, *1*.*60)*	*<* .*001*		*1*.*14 (0*.*80*, *1*.*62)*	.*733*	*0*.*82 (0*.*56*, *1*.*19)*	.*289*
More Than One	4/48(8.3)	0.37 (0.13, 1.02)	.055	6/ 59 (10.2)	1.41 (0.60, 3.28)	.430	1.25 (0.33, 4.69)	.746
*Adjusted*		*0*.*41 (0*.*14*, *1*.*17)*	.*094*		*2*.*42 (1*.*02*, *5*.*75)*	.*045*	*5*.*95 (1*.*52*, *23*.*25)*	.*011*
Unknown	71/357 (19.9)	1.00 (0.77, 1.31)	.989	17/216 (7.9)	1.06 (0.64, 1.75)	.816	0.34 (0.20, 0.60)	< .001
*Adjusted*		*1*.*10 (0*.*82*, *1*.*48)*	.*514*		*1*.*39 (0*.*79*, *2*.*43)*	.*257*	*1*.*26 (0*.*67*, *2*.*37)*	.*483*
**Ethnicity**								
Non-Hispanic (**REF**)	2231/11957 (18.7)	1.000		923/12321 (7.5)	1.00		0.35 (0.33, 0.38)	< .001
Hispanic	736/4022 (18.3)	0.97 (0.89, 1.07)	.612	71/1378 (5.2)	0.67 (0.52, 0.86)	.002	0.24 (0.19, 0.31)	< .001
*Adjusted*		*1*.*10 (0*.*97*, *1*.*26)*	.*144*		*0*.*85 (0*.*61*, *1*.*19)*	.*351*	*0*.*77 (0*.*54*, *1*.*11)*	.*161*
Unknown	216/ 774 (27.9)	1.68 (1.43, 1.99)	< .001	29/345 (8.4)	1.13 (0.77, 1.67)	.525	0.24 (0.16, 0.36)	< .001
*Adjusted*		*1*.*62 (1*.*34*, *1*.*94)*	*<* .*001*		*1*.*10 (0*.*72*, *1*.*70)*	.*658*	*0*.*68 (0*.*43*, *1*.*09)*	.*111*
**Insurance**								
Commercial **(REF)**	380/4318 (8.8)	1.00		184/4396 (4.2)	1.00		0.45 (0.38, 0.54)	< .001
Medicare	2423/9580 (25.3)	3.51 (3.13, 3.94)	< .001	665/6221 (10.7)	2.74 (2.32, 3.24)	< .001	0.35 (0.32, 0.39)	< .001
*Adjusted*		*1*.*41 (1*.*21*, *1*.*64)*	*<* .*001*		*1*.*51 (1*.*21*, *1*.*89)*	*<* .*001*	*1*.*08 (0*.*82*, *1*.*41)*	.*598*
Medicaid	230/1964 (11.7)	1.38 (1.16, 1.64)	< .001	76/1785 (4.3)	1.02 (0.78, 1.34)	.898	0.34 (0.26, 0.44)	< .001
*Adjusted*		*1*.*10 (0*.*92*, *1*.*32)*	.*293*		*1*.*00 (0*.*75*, *1*.*32)*	.*973*	*0*.*90 (0*.*65*, *1*.*26)*	.*550*
Uninsured	55/195 (28.2)	4.07 (2.93, 5.66)	< .001	22/434 (5.1)	1.22 (0.78, 1.92)	.386	0.14 (0.08, 0.23)	< .001
*Adjusted*		*2*.*90 (2*.*05*, *4*.*10)*	*<* .*001*		*1*.*19 (0*.*76*, *1*.*89)*	.*448*	*0*.*41 (0*.*23*, *0*.*73)*	.*002*
Other/Missing^b^	95/696 (13.6)	1.64 (1.29, 2.08)	< .001	76/1208 (6.3)	1.54 (1.17, 2.02)	.002	0.43 (0.39, 0.58)	< .001
*Adjusted*		*1*.*31 (1*.*02*, *1*.*68)*	.*033*		*1*.*42 (1*.*07*, *1*.*88)*	.*014*	*1*.*08 (0*.*74*, *1*.*57)*	.*678*
**BMI**								
Healthy Wt. **(REF**)	809/4174 (19.4)	1.00		170/2952 (5.8)	1.00		0.25 (0.21, 0.30)	< .001
Underweight	113/516 (21.9)	1.17 (0.93, 1.46)	.175	25/356 (7.0)	1.24 (0.80, 1.91)	.340	0.27 (0.17, 0.43)	< .001
*Adjusted*		*1*.*00 (0*.*79*, *1*.*25)*	.*972*		*1*.*11 (0*.*71*, *1*.*72)*	.*657*	*1*.*11 (0*.*68*, *1*.*82)*	.*682*
Overweight	946/5137 (18.4)	0.94 (0.85, 1.04)	.236	275/3948 (7.0)	1.23 (1.01, 1.49)	.044	0.33 (0.29, 0.38)	< .001
*Adjusted*		*1*.*13 (1*.*01*, *1*.*26)*	.*027*		*1*.*42 (1*.*16*, *1*.*74)*	.*001*	*1*.*26 (1*.*00*, *1*.*58)*	.*050*
Obese	940/5127 (18.3)	0.93 (0.84, 1.04)	.198	371/4860 (7.6)	1.35 (1.12, 1.63)	.002	0.37 (0.32, 0.42)	< .001
*Adjusted*		*1*.*45 (1*.*29*, *1*.*62)*	*<* .*001*		*1*.*84 (1*.*52*, *2*.*24)*	*<* .*001*	*1*.*27 (1*.*02*. *1*.*59)*	.*035*
Severely Obese	280/1513 (18.5)	0.95 (0.81, 1.10)	.458	164/1828 (9.0)	1.61 (1.29, 2.02)	< .001	0.43 (0.35, 0.53)	< .001
*Adjusted*		*2*.*02 (1*.*71*, *2*.*38)*	*<* .*001*		*2*.*71 (2*.*14*, *3*.*43)*	*<* .*001*	*1*.*34 (1*.*01*, *1*.*79)*	.*045*
Missing	95/286 (33.2)	2.07 (1.599, 2.677)	< .001	18/100 (18.0)	3.59 (2.11, 6.12)	< .001	0.44 (0.25, 0.78)	.005
*Adjusted*		*2*.*26 (1*.*72*, *2*.*96)*	*<* .*001*		*4*.*43 (2*.*56*, *7*.*69)*	*<* .*001*	*1*.*97 (1*.*06*, *3*.*64)*	.*031*
**Age**								
< 60 years (**REF**)	463/5958 (7.8)	1.00		300/7240 (4.1)	1.00		0.51 (0.44, 0.60)	< .001
60 to 70 years	796/4170 (19.1)	2.80 (2.48, 3.16)	< .001	284/3057 (9.3)	2.37 (2.03, 2.80)	< .001	0.43 (0.38, 0.50)	< .001
*Adjusted*		*2*.*61 (2*.*27*, *3*.*01)*	*<* .*001*		*1*.*99 (1*.*64*, *2*.*43)*	*<* .*001*	*0*.*76 (0*.*60*, *0*.*97)*	.*029*
> 70 years	1924/6625 (29.0)	4.86 (4.36, 5.42)	< .001	439/3747 (11.7)	3.07 (2.64, 3.58)	< .001	0.32 (0.29, 0.36)	< .001
*Adjusted*		*4*.*64 (3*.*98*, *5*.*42)*	*<* .*001*		*2*.*57 (2*.*05*, *3*.*23)*	*<* .*001*	*0*.*55 (0*.*42*, *0*.*73)*	*<* .*001*
**Total**	3183/16753 (19.0)			1023/14044 (7.3)			0.34	< .001
	95% CI: (.18 to .19)			95% CI: (.69 to .78)			95% CI: (0.311 to 0.361)	
*Total Adjusted*	*16*.*4%*			*9*.*2%*			*0*.*50*	*<* .*001*
	95% CI: (.*16 to* .*17)*			95% CI: (.*09 to* .*10)*			*95% CI*: *(0*.*367 to 0*.*673)*	

Note. Data for reference categories for each patient variable are italicized and underlined. Each of the other categories is compared with the reference category within each time period (within Period 1 or 2) for differences in mortality rate in comparison with the reference category within that time period. Reference categories are indicated by a bolded (‘**REF**’). These comparisons are reported both without adjustment and with adjustment for the Elixhauser score and the other patient variables (adjusted values are indicated by “Adjusted” in the left most column of relevant rows). For example, the adjusted model for sex accounts for race, ethnicity, insurance status, BMI, age, and the Elixhauser score. The two rightmost columns report the unadjusted and adjusted (in italicized rows) estimated odds ratios (ORs) for change in variable values from Period 1 to Period 2.

^a^Tests for the significance of change across the two time periods (February–April 2020 vs. July–September 2021) were in all cases performed using logistic regression estimates of odds ratios. When age, sex, race, ethnicity, insurance status, and BMI were each analyzed, the adjustments were performed using the remaining, other variables as covariates along with the Elixhauser score.

^b^Other Insurance Status: Combines VA insurance, TRICARE, Other State–Sponsored insurance, and other unspecified insurance.

Listed comparisons from Table 2 that had especially robust effects (with adjusted analyses at p’s < .01), shows the following comparison categories predicted higher mortality (vs. the reference category) in Period 1: male patients (vs. females); Asian patients (vs. White patients); patients on Medicare or uninsured (vs. commercial insurance); obese and severely obese patients (vs. healthy weight); and patients aged 60–70 years and over 70 (vs. < 60). In Period 1 the odds of death were over 4 times greater in those over 70, almost 3 times higher in the uninsured, and about double for the severely obese. In Period 2 only the following comparison categories had higher adjusted mortality rates: male patients (vs. females); patients on Medicare (vs. commercial insurance); severely obese patients (vs. healthy weight); and patients aged 60–70 years and >70 (vs. < 60). Thus, many of the patient groups having the highest mortality rates early in the pandemic did not have significantly elevated mortality rates at the end of the pandemic.

Table 2 displays, in the two rightmost columns, ORs for mortality change across time periods found within each comparison category both without and with adjustment for change in the other tabled patient variables (including Elixhauser).

The OR estimates without adjustment show that virtually all groups of patients experienced significant decreases in mortality across time, except for some relatively small groups such as American Indian and Pacific Islander patients and patients indicating more than one race. The OR estimates with adjustment (in italicized rows) show which groups sustained disproportionate changes in mortality (relative to the reference condition) when other covariates were controlled; a list of the categories showing especially robust decreases includes those listing more than one race (vs. White), uninsured patients (vs. commercial insurance), and patients who were over 70 (vs. < 60). Again, this shows a pattern consistent with the notion of the greatest improvements in mortality occurring in those who were initially at the highest risk.

Because of the limited availability of vaccines during the study period there were relatively few participants who reported positive vaccine status. However, including vaccine status amongst the covariates for the period of July to September 2021 showed that the effects of the patient level characteristics were little affected.

The continuous Elixhauser score is not shown amongst the patient variables in Table 2. However, the logistic regression-based relations of the Elixhauser with the three outcomes are presented in S13-S15 Figs in S1 File. These show that the Elixhauser was fairly strongly related to mortality and ICU admission, especially in Period 1. Its relations with intubation were negligible. S1 Table in S1 File presents the estimated coefficients and statistical significance of these relations in Periods 1 and 2.

## Discussion

This retrospective cohort study examined EHR data of 104,590 adult hospitalized patients with COVID from 21 U.S. health systems comprising a large diverse sample. Key COVID outcomes (rates of mortality, intubation, and ICU admission) were examined over the first 20 months of the pandemic, from February 1, 2020, to September 30, 2021.

The results show a marked decline in mortality rates, with mean unadjusted rates falling from 18.6% (95% CI: 18.0 to 19.2) in the first three months to 7.3% (95% CI: 6.9 to 7.8) in the last three months of the study period. Similar declines were seen with RSMR adjustment for age, sex, race, ethnicity, BMI, insurance status, and comorbidities. The greatest decline in mortality occurred early in the pandemic with modest increases in mortality occurring when hospital admissions increased (from November 2020 to January 2021 and from July 2021 to September 2021). Importantly, even though hospital admissions during the July to September 2021 peak exceeded levels seen early in the pandemic, mortality rates were about half of those seen early in the pandemic. These results agree with previous findings of a general improvement in COVID survivability over the course of the pandemic [2, 3, 5] but suggest that mortality improvements occurred primarily early and plateaued after the first several months. The initial decline in mortality rates likely reflects improvements in treatment and patient management [2, 3, 5, 19]. It is important to note that the above trends were found when data were collapsed across healthcare systems; thus, the patterns observed do not necessarily reflect the temporal association of admission and mortality rates that occurred in individual healthcare systems or hospitals. Other evidence gathered on hospitalized patients earlier in the pandemic (March-August 2020), suggests that late admission surges are associated with marked increases in mortality when examined on a per hospital basis [5]. In future analyses, the current data set will be used to examine the association of hospital admission and mortality at the level of the individual healthcare system.

ICU admissions rose substantially during periods when hospital admissions declined. The causes of such temporal association are unknown but warrant further study.

This research also found that improvement in mortality was generally present across all subpopulations of COVID patients examined in this study. However, adjusted mortality rates comparing the first and last 3 months of the study period suggested that some patient groups had significantly higher mortality rates across both periods relative to their reference condition: specifically, male patients (vs. females), those receiving Medicare (vs. commercial insurance), severely obese patients (vs. those of healthy weight), and patients 60 years of age and older (vs. those under 60). Other studies have found that male patients, the obese, and older patients are at heightened risk for severe COVID or COVID-related mortality [10, 20–22], so these results were precedented. However, receipt of Medicare insurance has not been consistently found to index increased risk of severe COVID or death [22]. Its significant association with mortality in the present study occurred despite covarying out the effects of age and comorbid conditions. It is possible that it was found in the current study because its large sample afforded relatively great statistical power.

Most patient subpopulations showed significant decreases in mortality over the two study periods, including: males; females; Asians; Blacks; Whites; Hispanics; non-Hispanics; patients having Medicare, Medicaid, and commercial insurance; uninsured patients; patients who were underweight, obese, and severely obese; and all three age groups (under 60, 60–70, > 70). Declines were not significant for some relatively small groups such as patients who were Pacific Islanders, American Indians, and those claiming more than one race. Analyses using statistically adjusted variables revealed groups that showed disproportionately greater declines (p < .01): uninsured patients showed greater declines than those with commercial insurance, and patients over 70 years of age showed greater declines than those under 60.

Some studies have found higher rates of mortality rates among Black and Hispanic COVID patients [10, 23–25] while others have not demonstrated that finding [26–28]. The present study found that Black patients had a significantly *lower* mortality rate than Whites during the first 3 months of the pandemic and Hispanics had a significantly *lower* mortality rate by the end of the study period (both with unadjusted rates). No differences were found amongst these groups when adjusted rates were analyzed. It is unknown why Black individuals suffered higher mortality rates in some studies and not the current one. The lack of a significant finding in the current study is unlikely due to inadequate statistical power. It may instead relate to the nature of the samples in the different studies. Most studies finding such a difference comprised both non-hospitalized and hospitalized persons [10, 23–25]. It may be that limiting the sample to hospitalized patients as in the current study restricts the range of illness severity in the sample and reduces the magnitudes of some associations. Some studies that have included a mix of hospitalized and non-hospitalized patients have not found racial differences in mortality [26], but these often were restricted with regard to sample sources and size. In sum, more research is needed to better understand how race is related to COVID severity.

### Strengths and limitations

Strengths of the study include the integration and analysis of EHR data from a very large cohort of hospitalized COVID patients admitted to 21 health systems from across the United States. The resulting data set is one of the largest assembled to date, providing a uniquely informative depiction of clinical trends over the course of the first 20 pandemic months, the patient characteristics associated with important clinical outcomes, and changes in these associations over time.

Several study limitations should also be considered. First, laboratory tests (other than COVID-19 PCR test results) and treatments used during hospitalization were not included in the current analyses and could have influenced the findings. Second, some health systems comprised multiple hospitals and, due to limitations in the data available, hospital-related effects could not be explored. Third, many of the 21 health systems included academic hospitals which could affect the representativeness of the results. Fourth, data on treatments or outcomes that occurred outside the health systems were not available. Fifth, differences in the number of patients coming from the various health systems over time might have affected the temporal patterns observed. Sixth, some patient subgroups could not be evaluated with adequate power given small sample sizes. Seventh, inconsistencies in the recording of discharge dispositions across health systems prevented consideration of discharge to hospice as a mortality equivalent. Subgroup comparisons on in-hospital mortality alone could be biased if there are disparities in the use of palliative care [29]. Finally, it is possible that some patients in this sample died from conditions that were unrelated to their COVID infection.

## Conclusions

This research has revealed marked improvements in mortality, intubation, and ICU admission rates across the first 20 months of the COVID pandemic amongst a sample of over 100,000 hospitalized patients with COVID, with only slight increases in mortality rates when COVID admissions rose markedly during pandemic surges occurring after the first few months of the pandemic (November 2020 to January 2021 and July to September 2021). Virtually all the different patient populations examined showed significant improvements in mortality rates over the course of the pandemic. However, some patient groups had relatively high mortality rates both early and late in the pandemic including males, those on Medicare, the severely obese, and those aged 60 and older. The findings highlight both overall progress in battling the COVID pandemic and populations that remain at heightened risk for negative outcomes. Finally, these findings suggest the need for additional research to identify clinical practices and treatments that contributed to the marked improvements in patient outcomes observed over time and that also attempts to identify other factors that influence differential treatment benefit across patient populations.

## Supporting information

S1 File Contains all the supporting tables and figures. (DOCX)
