# Supplementary material for: The first 20 months of the COVID-19 pandemic: Mortality, intubation and ICU rates among 104,590 patients hospitalized at 21 United States health systems
Source: PLoS One. 2022 Sep 28;17(9):e0274571. doi: 10.1371/journal.pone.0274571 (PMC9518859; doi:10.1371/journal.pone.0274571)
Supplement: S1 File — (DOCX) [file pone.0274571.s001.docx]

**Supporting Information**

Fiore et al. *The First 20 Months of the COVID-19 Pandemic: Mortality, Intubation and ICU Rates Among 104,590 Patients Hospitalized at 21 United States Health Systems*

**S1 Method. STROBE Checklist.**

**S2 Method: Inclusion Criteria for the COVID Electronic Health Record Cohort at the University of Wisconsin (CEC-UW) Total Sample and Analysis Sample.**

**S3 Method. Data Elements Extracted from the EHR.**

**S4 Method. Calculation of Body Mass Index (BMI).**

**S5 Method. Weighted Elixhauser Comorbidity Score Calculation.**

**S6 Method. Derivation of Risk Standardized Mortality Rates (RSMRs).**

**S1 Table. Estimated Coefficients and Statistical Significance of the Relations between Elixhauser Comorbidity Score and Mortality, Intubation, and ICU Admission Earlier (February-April 2020) and Later (July-September 2021) in the Pandemic.**

**S1 Fig. Geographic Map of CEC-UW Participating Health System Locations.**

**S2 Fig.** **Age Distribution for Hospitalized Patients at Each Study Month (March 2020 through September 2021).**

**S3 Fig. Elixhauser Comorbidity Score Distribution for Hospitalized Patients at Each Study Month (March 2020 through September 2021).**

**S4 Fig. Distribution of Length of Hospital Stay for Non-Deceased Patients at Each Study Month (March 2020 through September 2021).**

**S5 Fig. Unadjusted In-Hospital Mortality Rates (Deaths/Hospitalizations) and Number of Patients Hospitalized Per Month Across all 21 Health Systems.**

**S6 Fig. In-Hospital Mortality Rates (Deaths/Hospitalizations) and Number of Patients Hospitalized for the 21 Health Systems with RSMR Statistical Adjustment.**

**S7 Fig. Observed In-Hospital Mortality Rates (Deaths/Hospitalizations) and Number of Patients Hospitalized Per Month with Missing Data for Patient Race (March 2020 through September 2021).**

**S8 Fig. Observed In-Hospital Mortality Rates (Deaths/Hospitalizations) and Number of Patients Hospitalized Per Month with Missing Data for Patient Ethnicity (March 2020 through September 2021).**

**S9 Fig. Observed In-Hospital Mortality Rates (Deaths/Hospitalizations) and Number of Patients Hospitalized Per Month with Missing Data for Patient BMI (March 2020 through September 2021).**

**S10 Fig. Observed In-Hospital Mortality Rates (Deaths/Hospitalizations) and Number of Patients Hospitalized Per Month with Missing Data for Patient Insurance Status (March 2020 through September 2021).**

**S11 Fig. Adjusted In-Hospital Intubation Rates (Intubations/Hospitalizations) and Number of Patients Hospitalized Per Month Across All 21 Health Systems.**

**S12 Fig. Adjusted In-Hospital ICU Admission Rates (ICU Admissions/Hospitalizations) and Number of Patients Hospitalized Per Month Across All 21 Health Systems.**

**S13 Fig. Logistic Regression Based Relation of the Elixhauser Comorbidity Score with Mortality, Intubation Status, and ICU Admission Earlier (February-April 2020, Period 1) and Later (July-September 2021, Period 2) in the Pandemic.**

**S14 Fig. Logistic Regression Based Relation of the Elixhauser Comorbidity Score with Intubation Status Earlier (February-April 2020, Period 1) and Later (July-September 2021, Period 2) in the Pandemic.**

**S15 Fig. Logistic Regression Based Relation of the Elixhauser Comorbidity Score with ICU Admission Earlier (February-April 2020, Period 1) and Later (July-September 2021, Period 2) in the Pandemic.**

**References.**

This supplementary material has been provided by the authors to give readers additional information about their work.

**S1 Method.** Strobe Checklist.

STROBE Statement—Checklist of items that should be included in reports of ***cohort studies***

|  | Item No | Recommendation |  |
| --- | --- | --- | --- |
| **Title and abstract** | 1 | (*a*) Indicate the study’s design with a commonly used term in the title or the abstract | Abstract |
|  |  | (*b*) Provide in the abstract an informative and balanced summary of what was done and what was found | Abstract – Methods and Findings, Conclusions |
| Introduction | | |  |
| Background/rationale | 2 | Explain the scientific background and rationale for the investigation being reported | Introduction – Paragraphs 1-3 |
| Objectives | 3 | State specific objectives, including any prespecified hypotheses | Introduction – Paragraph 3 |
| Methods | | |  |
| Study design | 4 | Present key elements of study design early in the paper | Methods – Study design |
| Setting | 5 | Describe the setting, locations, and relevant dates, including periods of recruitment, exposure, follow-up, and data collection | Methods – Study population, Data collection |
| Participants | 6 | (*a*) Give the eligibility criteria, and the sources and methods of selection of participants. Describe methods of follow-up | Methods – Analysis sample; S2 Method |
|  |  | (*b*) For matched studies, give matching criteria and number of exposed and unexposed | N/A |
| Variables | 7 | Clearly define all outcomes, exposures, predictors, potential confounders, and effect modifiers. Give diagnostic criteria, if applicable | Methods – Primary and secondary outcomes, Non-outcome variables, Statistical analysis (Descriptive Statistics and Missingness, Changes in Outcomes Across the First 3 Months of the Study Period Compared to the Last 3 Months; Table 2) |
| Data sources/ measurement | 8* | For each variable of interest, give sources of data and details of methods of assessment (measurement). Describe comparability of assessment methods if there is more than one group | Methods – Data collection |
| Bias | 9 | Describe any efforts to address potential sources of bias | Methods – Primary and secondary outcomes, Non-outcome variables, Statistical analysis (Paragraphs 1-2),  Discussion - Limitations |
| Study size | 10 | Explain how the study size was arrived at | Methods – Analysis sample |
| Quantitative variables | 11 | Explain how quantitative variables were handled in the analyses. If applicable, describe which groupings were chosen and why | Methods – Analysis sample, Non-outcome variables, Statistical analysis |
| Statistical methods | 12 | (*a*) Describe all statistical methods, including those used to control for confounding | Methods – Statistical analysis |
|  |  | (*b*) Describe any methods used to examine subgroups and interactions | Methods – Statistical analysis |
|  |  | (*c*) Explain how missing data were addressed | Methods – Statistical analyses (Descriptive Statistics and Missingness) |
|  |  | (*d*) If applicable, explain how loss to follow-up was addressed | N/A |
|  |  | (*e*) Describe any sensitivity analyses | Results - Sensitivity analyses for change in mortality as a function of patient characteristics and health system |
| Results | | |  |
| Participants | 13* | (a) Report numbers of individuals at each stage of study—eg numbers potentially eligible, examined for eligibility, confirmed eligible, included in the study, completing follow-up, and analysed | Results (Paragraph 1, Characteristics of the participating health systems),  Fig 1 |
|  |  | (b) Give reasons for non-participation at each stage | Results (Paragraph 1), Fig 1 |
|  |  | (c) Consider use of a flow diagram | Fig 1 |
| Descriptive data | 14* | (a) Give characteristics of study participants (eg demographic, clinical, social) and information on exposures and potential confounders | Results – Characteristics of the participating health systems, Characteristics of analysis sample patients, Table 1 |
|  |  | (b) Indicate number of participants with missing data for each variable of interest | Results – Table 1 |
|  |  | (c) Summarise follow-up time (eg, average and total amount) | N/A |
| Outcome data | 15* | Report numbers of outcome events or summary measures over time | Results - Change in overall mortality, intubation rates, and ICU admission rates; Table 2; Change in mortality as a function of patient characteristics – all sections, Figs 2, 3a-b, 3c-d, 3e-f |
| Main results | 16 | (*a*) Give unadjusted estimates and, if applicable, confounder-adjusted estimates and their precision (eg, 95% confidence interval). Make clear which confounders were adjusted for and why they were included | Results - Unadjusted and risk standardized mortality rates, Change in overall mortality, intubation rates, and ICU admission rates; Table 2; Change in mortality as a function of patient characteristics (all sections)  Figs 2, 3a-b, 3c-d, 3e-f |
|  |  | (*b*) Report category boundaries when continuous variables were categorized | Methods - Additional non-outcome variables |
|  |  | (*c*) If relevant, consider translating estimates of relative risk into absolute risk for a meaningful time period | N/A |
| Other analyses | 17 | Report other analyses done—eg analyses of subgroups and interactions, and sensitivity analyses | Results - Unadjusted and risk standardized mortality rates, Sensitivity analyses for change in mortality as a function of patient characteristics and health system (Table 2)  Results - Associations between patient variables and mortality without and with the Inclusion of vaccination status  Results – Logistic regression-based relation of Elixhauser Comorbidity Score with mortality, intubation, and ICU admission earlier and later in the pandemic (S1 Table and S7-S10 Figs) |
| Discussion | | |  |
| Key results | 18 | Summarise key results with reference to study objectives | Discussion – Paragraphs 2-6 |
| Limitations | 19 | Discuss limitations of the study, taking into account sources of potential bias or imprecision. Discuss both direction and magnitude of any potential bias | Discussion - Limitations |
| Interpretation | 20 | Give a cautious overall interpretation of results considering objectives, limitations, multiplicity of analyses, results from similar studies, and other relevant evidence | Discussion – Paragraphs 8, Conclusion |
| Generalisability | 21 | Discuss the generalisability (external validity) of the study results | Discussion – Limitations |
| Other information | | |  |
| Funding | 22 | Give the source of funding and the role of the funders for the present study and, if applicable, for the original study on which the present article is based | Introduction (Paragraph 3)  Methods – Study Design  Methods - Statistical analysis (Statistical Analysis Plan) |

*Give information separately for exposed and unexposed groups.

**S2 Method.** Inclusion Criteria for the COVID Electronic Health Record Cohort at the University of Wisconsin (CEC-UW) Total Sample and Analysis Sample.

**Total Sample**: Criteria for inclusion in the CEC-UW project retrospective cohort were based on ICD-10 diagnoses and positive lab tests for COVID-19 that were evaluated based on electronic health record (EHR) data of patients in the 21 participating health systems from February 1, 2020, through September 30, 2021. Data extraction code was developed by CEC-UW project programmers and used by the 21 health systems to determine if a patient received either a COVID-19 diagnosis or a positive lab test result for COVID-19. Accordingly, the cohort for the CEC-UW total sample was defined as health system patients who met one or more of the following four inclusion criteria:

1. ICD-10 diagnosis of COVID-19 (U07.1 or J12.82).
2. COVID-19 polymerase chain reaction (PCR) test positive result.
3. COVID-19 antibody test positive result.
4. COVID-19 antigen test positive result.

A total of 1,154,612 patients met one or more of the criteria for inclusion in the CEC-UW retrospective cohort.

**Analysis Sample**: Criteria for inclusion in the current study of 104,590 hospitalized adult patients included:

1. Age 18 years or older.
2. The inpatient encounter must have been the first inpatient hospitalization and the duration of the inpatient encounter must have been at least 24 hours (or, if < 24 hours, admission to ICU or death during the hospitalization).
3. ICD-10 diagnosis of COVID-19 (U07.1 or J12.82) assigned during an initial inpatient encounter.
4. COVID-19 PCR test positive result within a 14-day time window starting 7 days prior to admission through the end of the first week of the initial inpatient encounter.
5. The patient must have had prior contact with the health system to permit extraction of pre-COVID-19 ICD-10 diagnoses for purposes of calculating the Elixhauser Comorbidity Score [1,2] (see **S5 Method**. Weighted Elixhauser Comorbidity Score Calculation).

**S3 Method.** Data Elements Extracted from the EHR.

Each data transfer included selected EHR information looking back to February 1, 2020, allowing for both the collection of information from new patients and follow-up data from individuals already in the cohort who received additional care at the health system. These retrospective updates were accomplished by assigning each patient in the data set from each health system an enduring cryptographically processed Patient ID based on the SHA256 algorithm, which yielded a 64-character unique and private hash-based message authentication code (HMAC). Secure transfer of data from each of the 21 health systems was accomplished via the transfer of data files to a secure SFTP (secure shell [SSH] File Transfer Protocol) portal located at the UW-Madison CEC-UW Coordinating Center. EHR data extraction code was provided via Github to all participating health systems with versions for Oracle-based and SQL-based EHR systems. Extraction code was further customized by the Information Technology (IT) staff at each of 21 health systems to account for health-system-specific EHR structures, tables, formats, and terminologies. The data extraction code was designed to locate and export data elements in a relatively uniform manner across the 21 health systems. The customization of the extraction code (i.e., with system-specific modifications) yielded partly harmonized data files for transfer to the CEC-UW Coordinating with the same basic set of data files and EHR variables within each data file, thus allowing later processing, additional harmonization, and ultimate merging of the data files across all 21 health systems according to a common set of specifications.

This data extraction process yielded five separate “source” data files from each of the 21 health systems formatted as comma-separated-values (CSV) files that were subsequently converted to SPSS data files at the CEC-UW Coordinating Center. Processing of the five source SPSS data files included preparation of variables for later analysis with appropriate formatting and labeling as well as computation of new variables such as body mass index (BMI) from weight and height. Further harmonization and merging of data across the 21 health systems were accomplished via computer programs developed by data management staff at the CEC-UW Coordinating Center.

Data elements (variables) were extracted from the EHR via specialized

programming code that created five data files containing groups of related data elements:

(1) The Patient Information data file is structured with one record per patient and includes health system ID, encrypted Patient ID, sex, age at COVID-19 diagnosis/positive test result, height, weight, race, ethnicity, insurance status, highest level of education, housing status, blood type, Rh Factor, 5-digit zip code of the patient, COVID-19 index date (the date that the patient was first diagnosed with COVID-19 or tested positive for COVID-19 via PCR, antibody, or antigen test), COVID-19 vaccine variables (vaccine name, manufacturer name, and date) for first and second doses, patient death date, and contact with health system prior to COVID-19 diagnosis/positive test result (yes/no). **In the current study, the patient Information variables used in analyses included: age, sex, race, ethnicity, body mass index (BMI; based on height and weight), insurance status, and vaccination status.** Vaccination status was coded ‘1’ if there was at least one non-missing vaccination dose date in the EHR, and ‘0’ if there were none. Whether the initial inpatient encounter occurred before the introduction of vaccines (on December 11, 2020) was also recorded in the Patient Information File.

(2) The Encounter-based data file is structured with one record per clinical encounter, but a patient could have multiple encounters (yielding multiple records per patient). Clinical encounters include inpatient hospital admissions, outpatient office visits, emergency department (ED) visits, urgent care visits, and other visits/contacts (e.g., telephone-based encounters; COVID-19 testing offered to patients in their cars in the hospital parking lot; etc.). Variables in the Encounter-based data file include health system ID, encrypted Patient ID, encrypted Encounter ID, encounter start day (relative to COVID-19 index date), encounter end day (relative to COVID-19 index date), encounter sequence, treatment site (inpatient, outpatient office visit equivalent, ED, urgent care, and other), zip code of treatment site, encounter type (text field denoting the participant-specific encounter type for the encounter), ICD-10 diagnoses assigned during the encounter (based on fields in the EHR for admission, discharge, and encounter diagnoses), ICU admission (yes/no), required supplemental oxygen (yes/no), non-invasive positive pressure (yes/no), intubated for ventilator use (yes/no), pneumonia (yes/no), septic shock (yes, no),number of days hospitalized, mortality during admission (yes/no), smoking tobacco status (current, former, never), smoking status day reference (days from encounter day that the smoking status was recorded), smokeless tobacco status, type of tobacco (cigarettes, pipe, cigars, snuff, chew), e-cigarettes/electronic nicotine delivery systems, tobacco use years, pack years, years since quitting, packs per day, marijuana use, passive smoke exposure, signs/symptoms on presentation including fever >100.4F, initial pulse rate, initial systolic BP, initial diastolic BP, oxygen saturation level, chills, muscle aches (myalgia), runny nose (rhinorrhea), sore throat, cough (new onset or worsening of chronic cough), shortness of breath (dyspnea), nausea or vomiting, headache, abdominal pain, diarrhea, dizziness, impaired consciousness, acute cerebrovascular disease (stroke and TIA), ataxia, seizure, taste impairment, smell impairment, vision impairment, nerve pain, and skeletal muscular pain. **In the current study, only the following Encounter-based variables were used in analyses: COVID-19 diagnosis (yes/no), mortality during admission (yes/no), intubated for ventilator use (yes/no), and ICU admission (yes/no). COVID-19 diagnosis was based on assignment of U07.1 or J12.82 codes during the initial inpatient encounter.**

(3) The ICD-10 Diagnosis data file includes variables that capture all ICD-10 codes prior to and after a COVID-19 diagnosis for a patient including health system ID, encrypted Patient ID, and the following three variables for each unique ICD-10 code assigned to the patient: the count of ICD-10 diagnoses, the first date on which the ICD-10 diagnosis was recorded, and the last date on which this diagnosis was recorded. The data file includes four versions of these latter three variables: from any source in the EHR, from the admission diagnosis field in the EHR, from the discharge diagnosis field in the EHR, and from the encounter diagnosis field in the EHR. Typically, only inpatient encounters had admission and discharge diagnoses whereas all encounter types (inpatient, outpatient office visit equivalent, ED, urgent care, and other) had “encounter” diagnoses. **The pre-COVID ICD-10 diagnoses were used to compute the van Walraven weighted Elixhauser Comorbidity Score, [1,2] based on diagnoses made in the past 5 years (prior to a patient getting COVID-19).** (For detailed information on the Elixhauser Comorbidity Score, see **S5 Method**).

(4) The Laboratory Test data file is structured with one record per lab test for a given patient, but the patient could have multiple types of lab tests (yielding multiple records per patient) with each type of lab test potentially being ordered multiple times. Variables in the Laboratory Test data file include health system ID, encrypted Patient ID, encrypted Encounter ID, lab test order day (relative to index date), lab test result day (relative to index date), lab sequence (sequential ordering of lab tests for the patient over time), lab test name, LOINC code (see details below in the section on *Processing of Laboratory Test Data*), lab result (numeric or character data such as “detected” or “negative”), and unit of measure for the numeric lab test (e.g., mg/dL). Lab tests included COVID-19 tests (SARS-CoV-2 PCR, SARS-CoV-2 IGM antigen test, SARS-CoV-2 IGG antibody test), hematology (white blood cell count, absolute neutrophil, absolute lymphocyte, hemoglobin, hematocrit, platelet count), chemistries (sodium, potassium, chloride, bicarbonate, blood urea nitrogen, creatinine, glucose, calcium), liver function (total protein, albumin, aspartate aminotransferase, alanine aminotransferase, total bilirubin, alkaline phosphatase), coagulation (international normalized ratio, d-dimer), inflammatory markers (erythrocyte sedimentation rate, c-reactive protein, ferritin, lactate dehydrogenase, procalcitonin, interleukin-6), cardiovascular (troponin), diabetes (hemoglobin A1c), and infectious studies (respiratory PCR panel, influenza PCR, and influenza). **In the current study, only the following variables from the** **Laboratory Tests data file were used in the analyses: the COVID-19 PCR, antibody, and antigen tests were used for cohort eligibility determination. The COVID-19 PCR test was also used in the determination of the analysis sample of adult hospitalized patients with COVID-19.**

(5) The Medications data file is structured with one record per medication order for a given patient, but a patient could have multiple types of medications ordered (yielding multiple records per patient) with each type of medication potentially being ordered multiple times. Variables in the Medications data file include health system ID, encrypted Patient ID, encrypted Encounter ID, medication order day (relative to index date), medication order sequence (sequential ordering of medications for the patient over time); medication name, and RxNorm code (see details below in the section on *Processing of Medication Data*). Individual medications from the following 10 medication classes were extracted from the EHR: blood thinners, steroids, ace inhibitors, angiotensin receptor blockers (ARBs), adrenergic bronchodilators (short acting + long acting), anticholinergic bronchodilators, bronchodilator combinations, inhaled corticosteroids (including combo medications), tobacco cessation medications, and medications used to treat COVID-19 (ascorbic acid, chloroquine, hydroxychloroquine, remdesivir, tocilizumab, covid convalescent plasma, baricitinib, regeneron ab, casirivimab, imdevimab, bamlanivimab, sarilumab, molnupiravir, nirmatrelvir, nirmatrelvir/ritonavir combo, paxlovid [branded nirmatrelvir/ritonavir], evusheld [tixagevimab/cilgavimab combo], tixagevimab, cilgavimab, and etesevimab). **In the current study, variables from the** **Medications data file were not used in the analyses.**

*Processing of Laboratory Test Data*

As noted above, the Laboratory Test data file contains both lab test names as well as LOINC (Logical Observation Identifiers, Names and Codes) codes. The LOINC system is a database of terms and standards for identifying medical laboratory tests and results and is maintained and distributed by the Regenstrief Institute, Inc. LOINC codes are used by CDC, Veterans Affairs – Department of Defense (VA-DoD), laboratory services providers, insurance companies, researchers, and many healthcare systems in the United States. More information on LOINC codes can be found at: <https://www.regenstrief.org/implementation/loinc/> .

LOINC codes are unique codes that indicate the following:

- Component - what is being measured (e.g., “PLATELET COUNT “; “CORONAVIRUS (COVID-19) SARS-COV-2”)
- Property - volume, mass, length, etc.
- Time - interval of time over which the observation or measurement was made
- System – specimen type, e.g., blood, urine, etc.
- Scale – unit of measurement
- Method - procedure used to make the measurement or observation

In the CEC-UW project, data extraction code captured Lab Test Name, result, and unit of measure for the result (if applicable) for selected lab tests for all 21 participating health systems; LOINC codes were collected for most health systems (only a few do not use LOINC codes). Lab test names, LOINC codes, and results are processed via programming at the CEC-UW Coordinating Center that produces the harmonized test result variables for each patient lab test record across the 21 health systems. The resulting variables are date-stamped for merging with other data. Reference ranges for most tests were based on *ABIM (American Board of Internal Medicine) Laboratory Test Reference Ranges* available at <https://www.abim.org/Media/bfijryql/laboratory-reference-ranges.pdf>.

*Processing of Medication Data*

As noted above, the Medications data file contains both medication names as well as RxNorm codes. RxNorm is a standardized naming system to codify clinical drugs and is part of the Federal Medication Terminologies initiative; RxNorm is maintained and distributed by the National Library of Medicine. There are RxNorm codes for generic and branded versions of clinical medications and these codes are used by the Center for Medicare and Medicaid Services (CMS), Veterans Affairs – Department of Defense (VA-DoD), insurance companies, pharmacies, researchers, and many healthcare systems in the United States. RxNorm codes represent unique numeric codes for each version of generic and branded medications based on ingredients, strength, and dose form. More information on RxNorm codes can be found at: <https://www.nlm.nih.gov/research/umls/rxnorm/index.html> .

In the CEC-UW project, data extraction code captures medication names for selected medications for all 21 participating health systems; RxNorm codes were collected for most health systems (only a few do not use RxNorm codes). Medication names and RxNorm codes were processed via programming at the CEC-UW Coordinating Center that grouped together under a common name all generic and branded medications for each category of medications (e.g., blood thinners, steroids, etc.) without regard to the specific dose or strength similarly. The resulting variables are date-stamped for merging with other data.

**S4 Method.** Calculation of Body Mass Index (BMI).

The Analysis Sample consists of hospitalized COVID-19 patients aged 18 years or older. For patients ≥20 years of age, BMI was calculated as: [weight (lb) / height^2^ (in)] x 703. Calculation of BMI for patients younger than 20 years of age (i.e., 18 and 19 years old in the Analysis Sample) required specialized processing with a SAS program based on Centers for Disease Control and Prevention (CDC) growth charts (program: “cdc-source-code.sas”; available at: <https://www.cdc.gov/nccdphp/dnpao/growthcharts/resources/sas.htm>). This program requires patient age in months, sex, height (cm), and weight (kg), all of which are part of the Patient Information data file. For input to the SAS program, the four patient variables were written to a plain text (ASCII). Results of the SAS program include z-scores, percentiles, and flags for extreme values; these values were written to a SAS dataset that was imported into SPSS for processing and merging with other Patient Information variables. BMI percentiles were then converted into BMI categories based on CDC cutoffs at <https://www.cdc.gov/obesity/childhood/defining.html> :

- Underweight: Less than the 5th percentile
- Normal or Healthy Weight: 5th percentile to less than the 85th percentile
- Overweight: 85th to less than the 95th percentile
- Obese: 95th percentile to less than 99th percentile
- Severely obese 99th percentile or greater

**S5 Method.** Weighted Elixhauser Comorbidity Score Calculation.

Calculation of the Elixhauser Comorbidity Score [1] used ICD-10 diagnoses from the EHR with a 5-year look back pre-COVID-19. The diagnostic groups were identified by using the ICD-10 diagnosis codes listed in Quan et al. [3] The coding algorithm scans patient diagnostic codes and identifies whether a given ICD-10 code belongs to one or more of 31 different Elixhauser Comorbidity Groups (see Table below). Each of the Comorbidity Groups is coded as a binary variable where a value of 1 indicates the comorbid condition is present and a value of 0 indicates that the comorbid condition is absent. The total Elixhauser Comorbidity Score is a weighted sum of the 31 binary comorbid conditions, with the weights based on van Walraven et al [2] (see Table below). The original SAS code for this program was developed by Quan et al [3] at the University of Calgary, Manitoba Centre for Health Policy and modified by CEC-UW data management staff.

| **Calculation of Weighted Elixhauser Comorbidity Score** | | |
| --- | --- | --- |
| **Comorbidity Group** | **ICD-10 codes** | **van Walraven**  **weights** |
| Congestive heart failure | I09.9, I11.0, I13.0, I13.2, I25.5, I42.0, I42.5 - I42.9, I43.x, I50.x, P29.0 | 7 |
| Cardiac arrhythmias | I44.1 - I44.3, I45.6, I45.9, I47.x - I49.x, R00.0, R00.1, R00.8, T82.1, Z45.0, Z95.0 | 5 |
| Valvular disease | A52.0, I05.x - I08.x, I09.1, I09.8, I34.x - I39.x, Q23.0 - Q23.3, Z95.2 - Z95.4 | -1 |
| Pulmonary circulation disorders | I26.x, I27.x, I28.0, I28.8, I28.9 | 4 |
| Peripheral vascular disorders | I70.x, I71.x, I73.1, I73.8, I73.9, I77.1, I79.0, I79.2, K55.1, K55.8, K55.9, Z95.8, Z95.9 | 2 |
| Hypertension, uncomplicated | I10.x | 0 |
| Hypertension, complicated | I11.x - I13.x, I15.x | 0 |
| Paralysis | G04.1, G11.4, G80.1, G80.2, G81.x, G82.x, G83.0 - G83.4, G83.9 | 7 |
| Other neurological disorders | G10.x - G13.x, G20.x - G22.x, G25.4, G25.5, G31.2, G31.8, G31.9, G32.x, G35.x - G37.x, G40.x, G41.x, G93.1, G93.4, R47.0, R56.x | 6 |
| Chronic pulmonary disease | I27.8, I27.9, J40.x - J47.x, J60.x - J67.x, J68.4, J70.1, J70.3 | 3 |
| Diabetes, uncomplicated | E10.0, E10.1, E10.9, E11.0, E11.1, E11.9, E12.0, E12.1, E12.9, E13.0, E13.1, E13.9, E14.0, E14.1, E14.9 | 0 |
| Diabetes, complicated | E10.2 - E10.8, E11.2 - E11.8, E12.2 - E12.8, E13.2 - E13.8, E14.2 - E14.8 | 0 |
| Hypothyroidism | E00.x - E03.x, E89.0 | 0 |
| Renal failure | I12.0, I13.1, N18.x, N19.x, N25.0, Z49.0 - Z49.2, Z94.0, Z99.2 | 5 |
| Liver disease | B18.x, I85.x, I86.4, I98.2, K70.x, K71.1, K71.3 - K71.5, K71.7, K72.x - K74.x, K76.0, K76.2 - K76.9, Z94.4 | 11 |
| Peptic ulcer disease, excluding bleeding | K25.7, K25.9, K26.7, K26.9, K27.7, K27.9, K28.7, K28.9 | 0 |
| AIDS/HIV | B20.x - B22.x, B24.x | 0 |
| Lymphoma | C81.x - C85.x, C88.x, C96.x, C90.0, C90.2 | 9 |
| Metastatic cancer | C77.x - C80.x | 12 |
| Solid tumour without metastasis | C00.x - C26.x, C30.x - C34.x, C37.x - C41.x, C43.x, C45.x - C58.x, C60.x - C76.x, C97.x | 4 |
| Rheumatoid arthritis/collagen vascular diseases | L94.0, L94.1, L94.3, M05.x, M06.x, M08.x, M12.0, M12.3, M30.x, M31.0 - M31.3, M32.x - M35.x, M45.x, M46.1, M46.8, M46.9 | 0 |
| Coagulopathy: | D65 - D68.x, D69.1, D69.3 - D69.6 | 3 |
| Obesity | E66.x | -4 |
| Weight loss | E40.x - E46.x, R63.4, R64 | 6 |
| Fluid and electrolyte disorders | E22.2, E86.x, E87.x | 5 |
| Blood loss anaemia | D50.0 | -2 |
| Deficiency anaemia | D50.8, D50.9, D51.x - D53.x | -2 |
| Alcohol abuse | F10, E52, G62.1, I42.6, K29.2, K70.0, K70.3, K70.9, T51.x, Z50.2, Z71.4, Z72.1 | 0 |
| Drug abuse | F11.x - F16.x, F18.x, F19.x, Z71.5, Z72.2 | -7 |
| Psychoses | F20.x, F22.x - F25.x, F28.x, F29.x, F30.2, F31.2, F31.5 | 0 |
| Depression | F20.4, F31.3 - F31.5, F32.x, F33.x, F34.1, F41.2, F43.2 | -3 |

**S6 Method.** Derivation of Risk Standardized Mortality Rates (RSMRs).

We applied a generalized linear mixed modeling (GLMM) framework involving nesting of COVID-19 patient (j) within health system (i), with patient-level mortality ($Y_{ij}$=0 implying no death; 1=death) as outcome and patient-characteristics as covariates (X). We used the lme4 R package:

(<https://cran.r-project.org/web/packages/lme4/index.html>) for model estimation. The rationale for assessing outcomes while controlling for the covariates (the seven covariates [age, sex, race, ethnicity, BMI, insurance status, and Elixhauser Comorbidity Score] and health system) is that such control calibrated outcomes across health systems and months. These comparisons reflect time course differences with the influence of the covariates removed, which may change in distribution across health systems/month.

In calculating RSMR for health systems, to account for potential variability in covariate effects over time, we conducted separate analyses by month (February 2020 – September 2021). The GLMM can be written as:

$$logit\Pr\left( Y_{ij}=1 \right)= \beta_{0}+\sum_{k=1}^{K} X_{ijk}\beta_{k}+b_{i0}$$

where $\beta_{0}$ denotes a fixed intercept, $\sum_{k=1}^{K} X_{ijk}\beta_{k}$ represents the covariate adjustment, and $b_{i0}$ represents the system-specific random effect, assumed to follow a Gaussian distribution with mean 0, variance $\tau_{0}^{2}$. The system-specific random effect represents the overall mortality associated with each system compared to the 21 health systems after controlling for the effects of patient-level covariates.

To obtain risk-adjusted scores for each system, we obtained the health system specific predicted score for each patient as:

$p_{ij}=E\left( Y_{ij}=1 \right|X_{ij},b_{i0}) =1/\{1+\exp\left( -\beta_{0}-\sum_{k=1}^{K} X_{ijk}\beta_{k}-b_{i0} \right)\}$,

We in turn directly derived risk standardized mortality rates (RSMRs) by averaging the mortality rates assuming that each patient was hypothetically treated within each of the 21 health systems. Following Asch et al.’s [4] notation, if $i^{*}$ denotes a hypothetical system in which patient *j* is treated, we obtain patient j’s estimate for each of the $i^{*}$=1, …, 21 systems:

$p_{i^{*}j}=E\left( Y_{i^{*}j}=1 \right|X_{i^{*}j},b_{i^{*}0}) =1/\{1+\exp\left( -\beta_{0}-\sum_{k=1}^{K} X_{ijk}\beta_{k}-b_{i^{*}0} \right)\}$.

These quantities are invoked in defining directly standardized event rates for each system as:

$$s_{i,DS}=\sum_{i^{*}=1}^{21} \sum_{j=1}^{n_{i^{*}}} p_{i^{*}j}/N$$

where the$p_{i^{*}j}$s are consistently calculated using the estimated $b_{i0}$ for system *i.*

The results are depicted in S3 Fig. Thus, S3 Fig shows each health system’s mortality rate adjusted for how its patient characteristics differed from the characteristics of patients at other health systems within that month. In Figure 2, S5 Fig and S6 Fig the same basic GLMM approach was applied to data across all months (pooling health systems) to adjust for changes in aggregate patient characteristics across time. Patients were viewed as nested within month, with each month having its own random effect. As with the health system RMSRs, the month effects were converted to proportions by averaging the mortality rates assuming that each patient was hypothetically treated within each of the 20 months.

**S1 Table.** Estimated Coefficients and Statistical Significance of the Relations between Elixhauser Comorbidity Score and Mortality, Intubation, and ICU Admission Earlier (February-April 2020) and Later (July-September 2021) in the Pandemic.

| **Mortality** | | | | | | | | |
| --- | --- | --- | --- | --- | --- | --- | --- | --- |
| **Period** | **Coefficient** | **B** | **SE** | **Wald** | **df** | **p-value** | **Exp(B)** | **95% CI** |
| Feb-Apr, 2020 | Elixhauser | .025 | .002 | 139.97 | 1 | <.001 | 1.03 | 1.02, 1.03 |
|  | Intercept | -1.569 | .023 | 4771.93 | 1 | <.001 | 0.21 |  |
| July-Sep, 2021 | Elixhauser | .024 | .003 | 73.61 | 1 | <.001 | 1.03 | 1.02, 1.03 |
|  | Intercept | -2.701 | .039 | 4780.19 | 1 | <.001 | 0.07 |  |
|  | | | | | | | | |
| **Intubation** | | | | | | | | |
| **Period** | **Coefficient** | **B** | **SE** | **Wald** | **df** | **p-value** | **Exp(B)** | **95% CI** |
| Feb-Apr, 2020 | Elixhauser | -.005 | .002 | 3.89 | 1 | 0.049 | 1.00 | 0.99, 1.00 |
|  | Intercept | -1.32 | .021 | 3798.95 | 1 | <.001 | 0.27 |  |
| July-Sep, 2021 | Elixhauser | .002 | .003 | 0.33 | 1 | 0.577 | 1.00 | 0.01, 1.01 |
|  | Intercept | -2.01 | .031 | 4187.79 | 1 | <.001 | 0.13 |  |
|  | | | | | | | | |
| **ICU Admission** | | | | | | | | |
| **Period** | **Coefficient** | **B** | **SE** | **Wald** | **df** | **p-value** | **Exp(B)** | **95% CI** |
| Feb-Apr, 2020 | Elixhauser | .017 | .002 | 68.73 | 1 | <.001 | 1.02 | 1.01, 1.02 |
|  | Intercept | -1.40 | .022 | 4136.24 | 1 | <.001 | 0.25 |  |
| July-Sep, 2021 | Elixhauser | .007 | .002 | 9.69 | 1 | 0.002 | 1.01 | 1.00, 1.01 |
|  | Intercept | -1.34 | .025 | 2950.15 | 1 | <.001 | 0.26 |  |

**S1 Fig.** Geographic Map of CEC-UW Participating Health System Locations


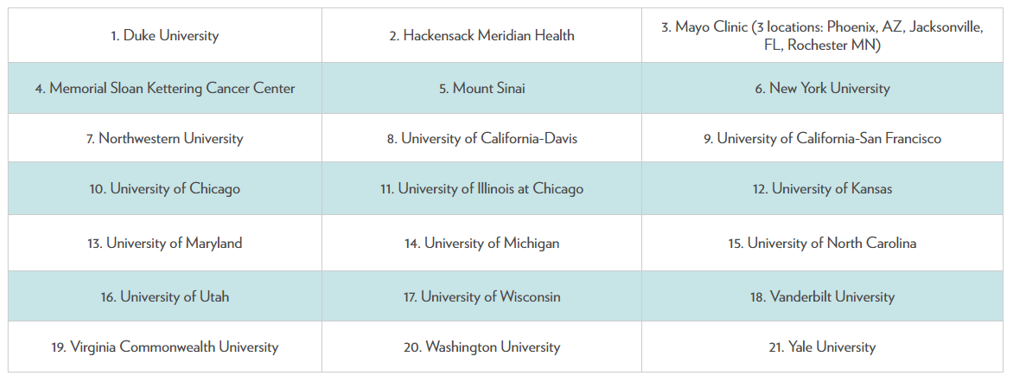

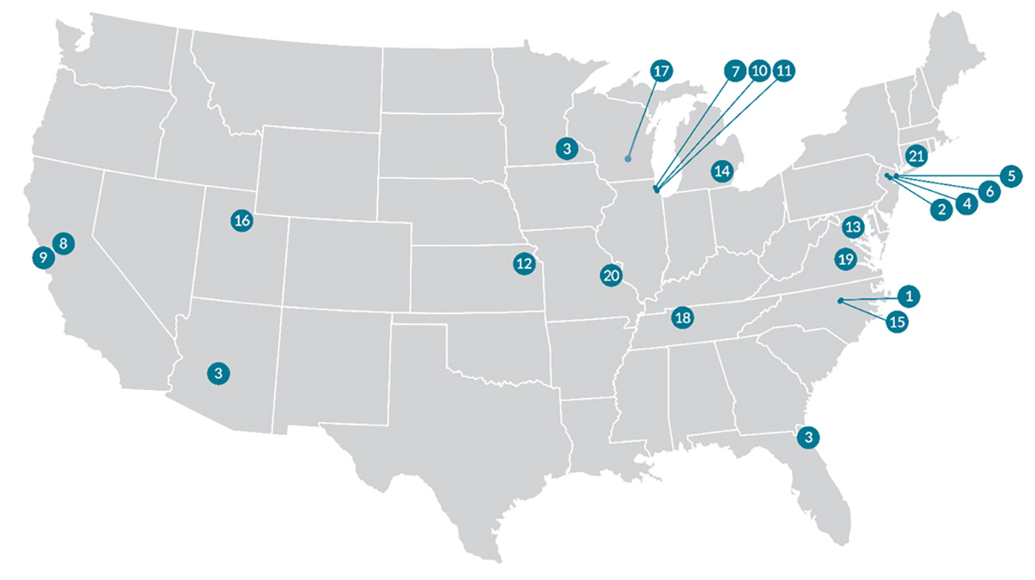


Republished from reference 5 under a CC BY license, with permission from Kay Lera, original copyright 2020.

**S2 Fig.** Age Distribution for Hospitalized Patients at Each Study Month (March 2020 through September 2021).

**
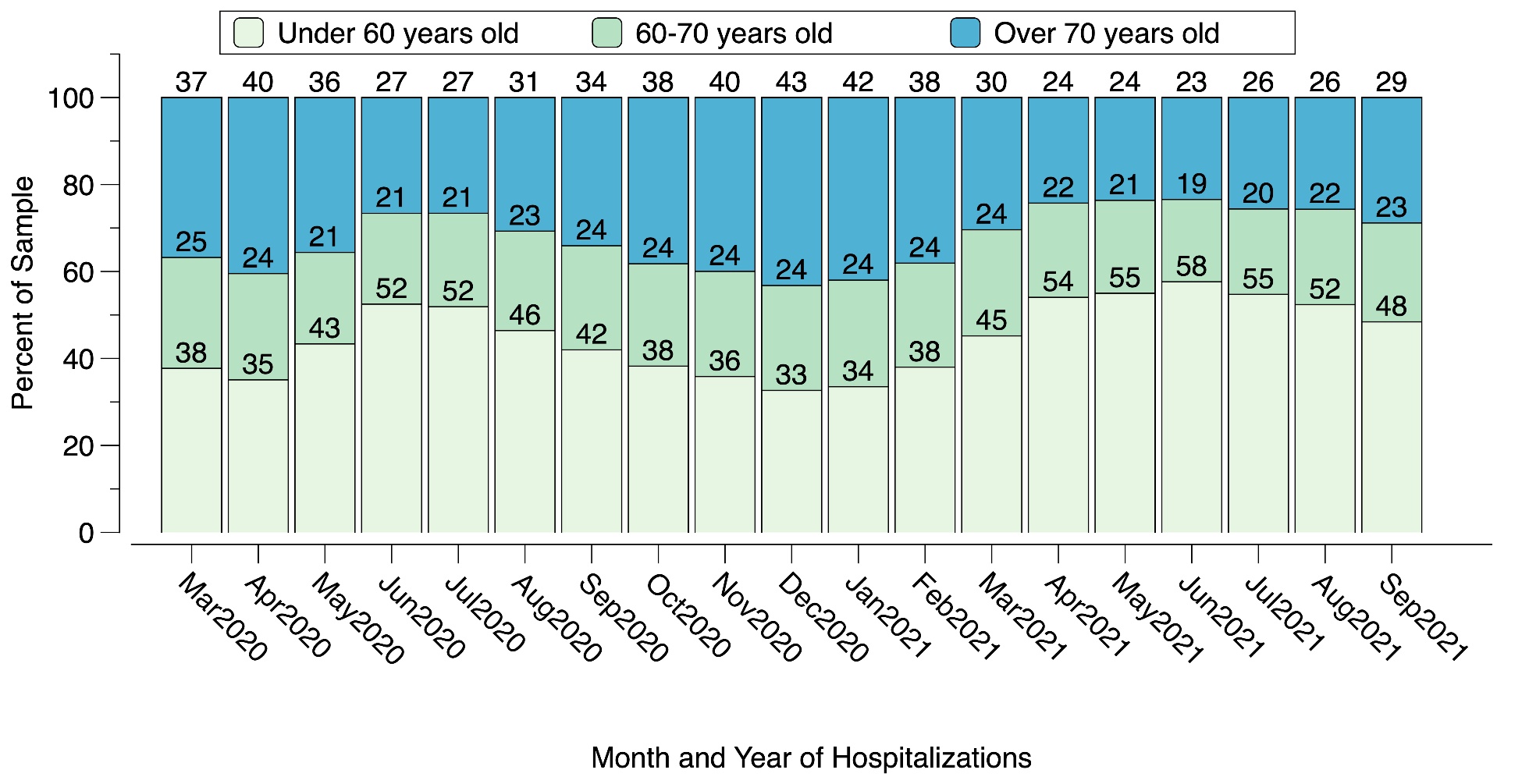
**

**S3 Fig.** Elixhauser Comorbidity Score Distribution for Hospitalized Patients at Each Study Month (March 2020 through September 2021).

**
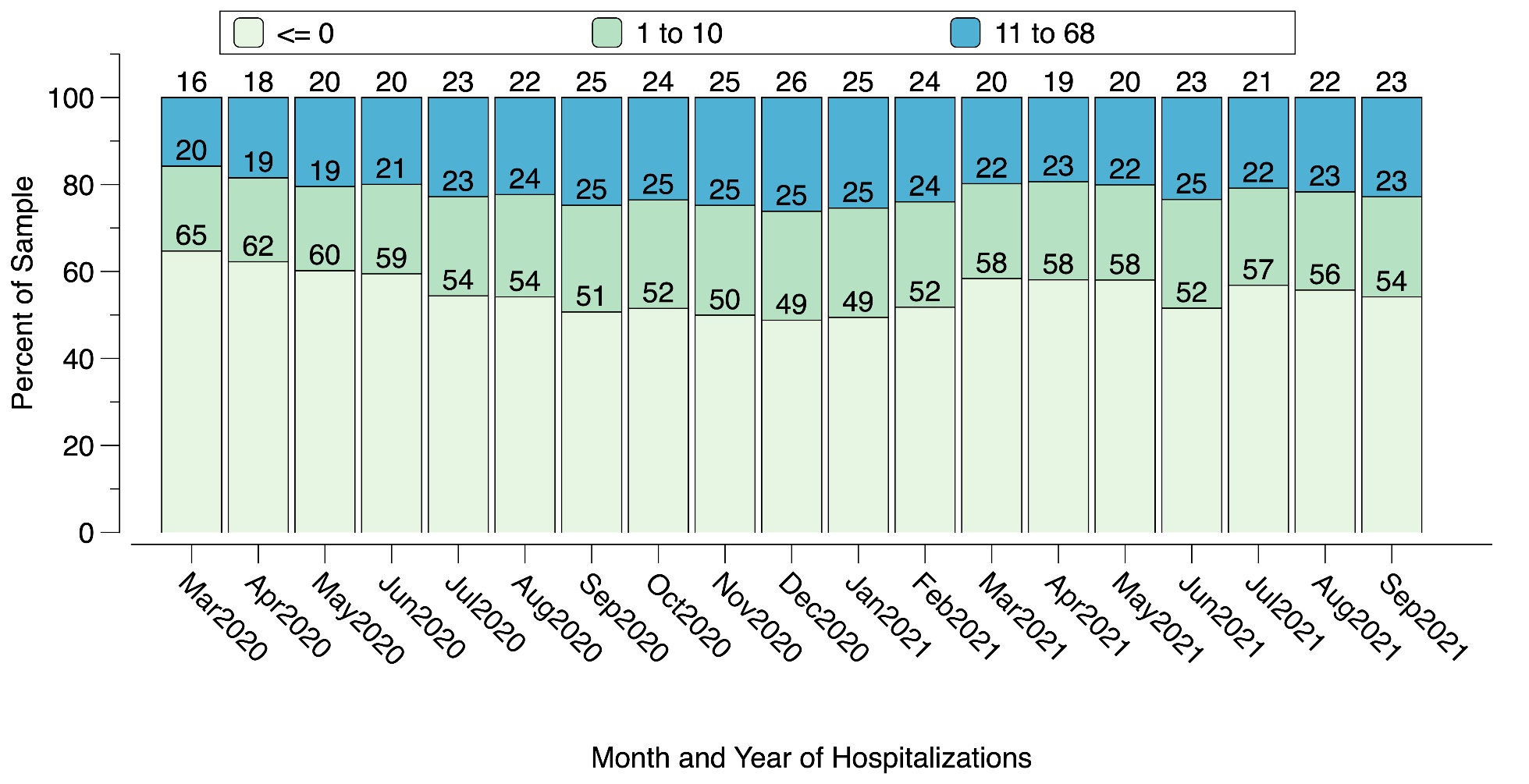
**

**S4 Fig.** Distribution of Length of Hospital Stay for Non-Deceased Patients at Each Study Month (March 2020 through September 2021).

**
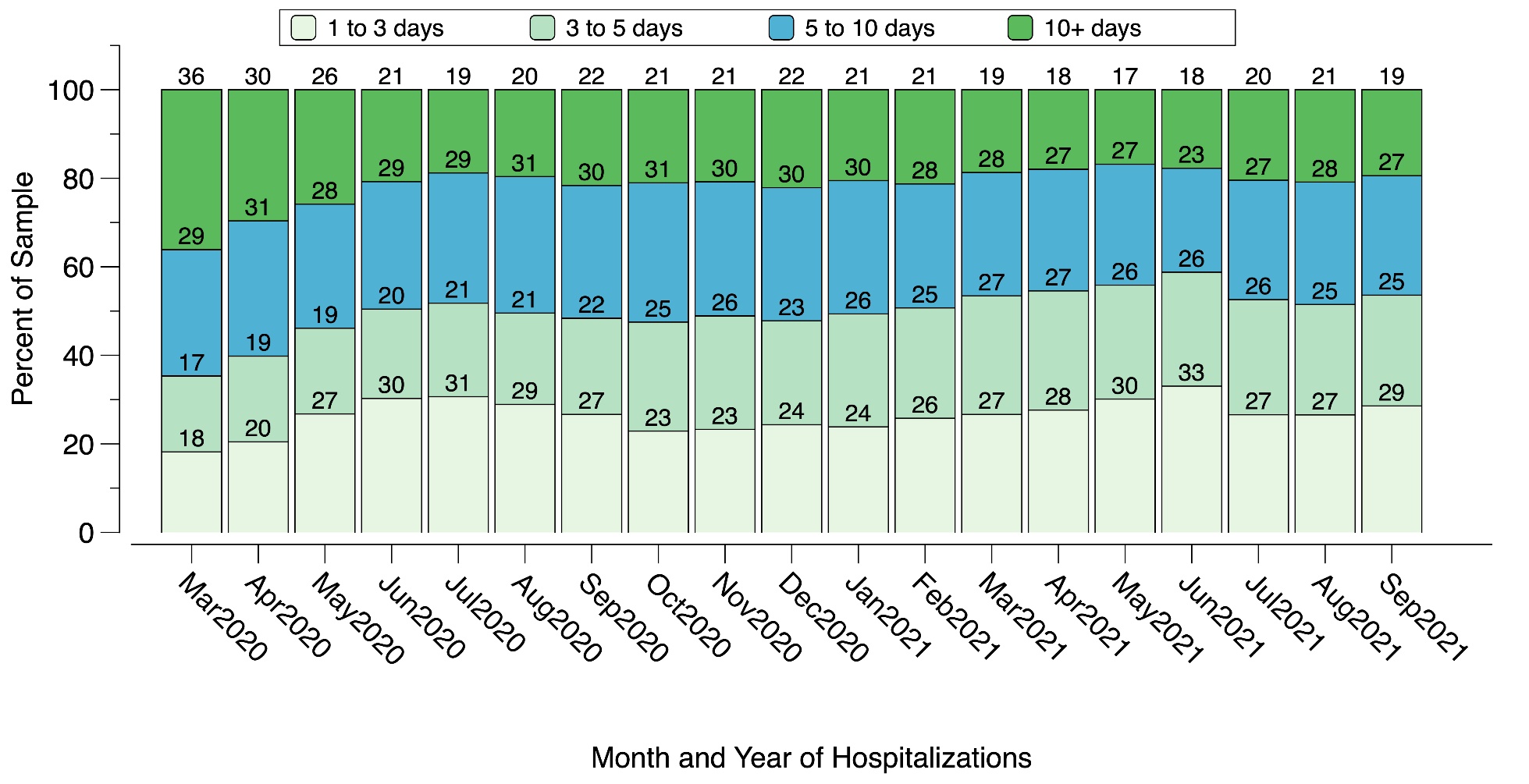
**

**S5 Fig.** Unadjusted In-Hospital Mortality Rates (Deaths/Hospitalizations) and Number of Patients Hospitalized Per Month Across all 21 Health Systems.

**
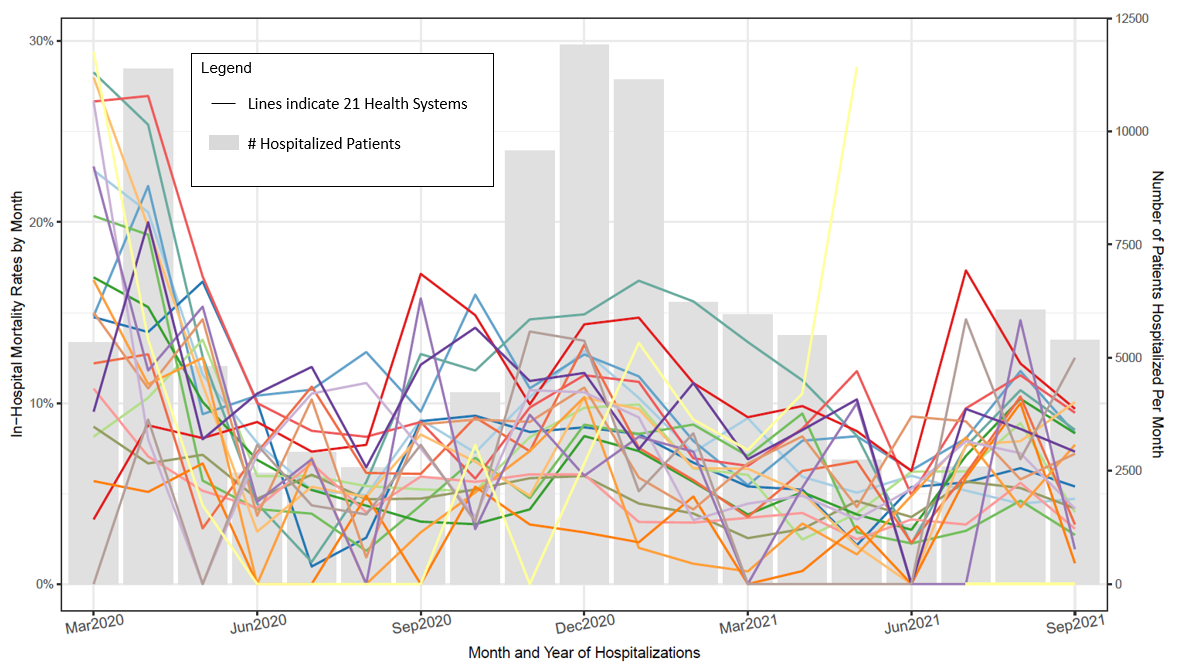
**

**S6 Fig.** In-Hospital Mortality Rates (Deaths/Hospitalizations) and Number of Patients Hospitalized for the 21 Health Systems with RSMR Statistical Adjustment.


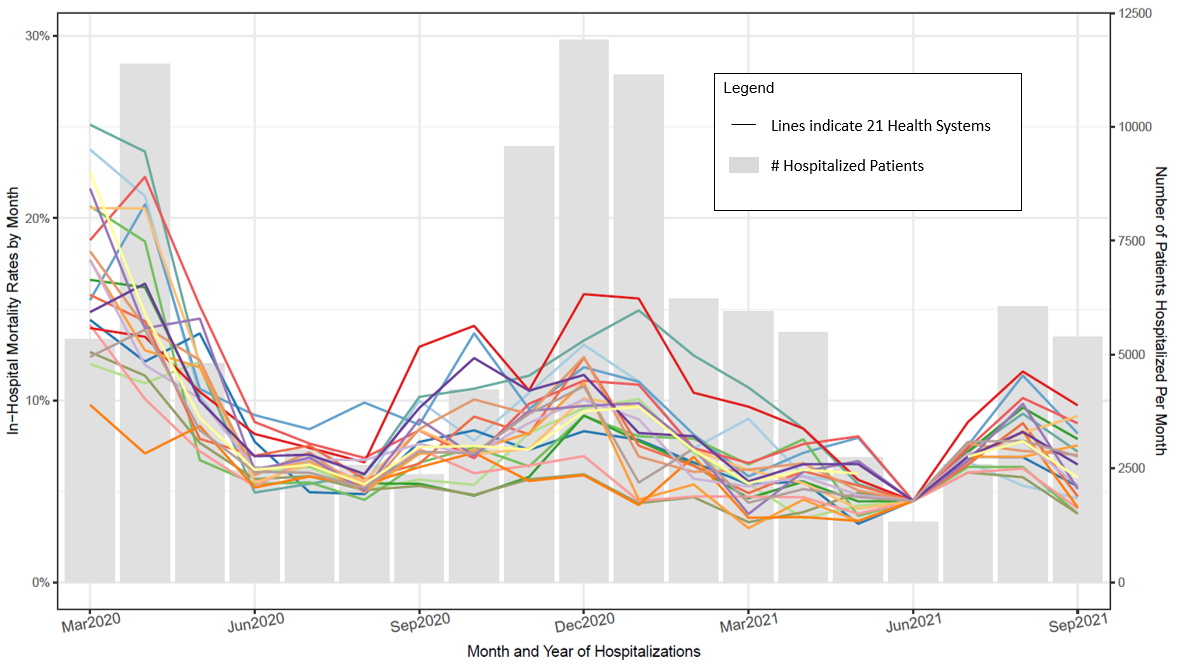


**S7 Fig.** Observed In-Hospital Mortality Rates (Deaths/Hospitalizations) and Number of Patients Hospitalized Per Month with Missing Data for Patient Race (March 2020 through September 2021).


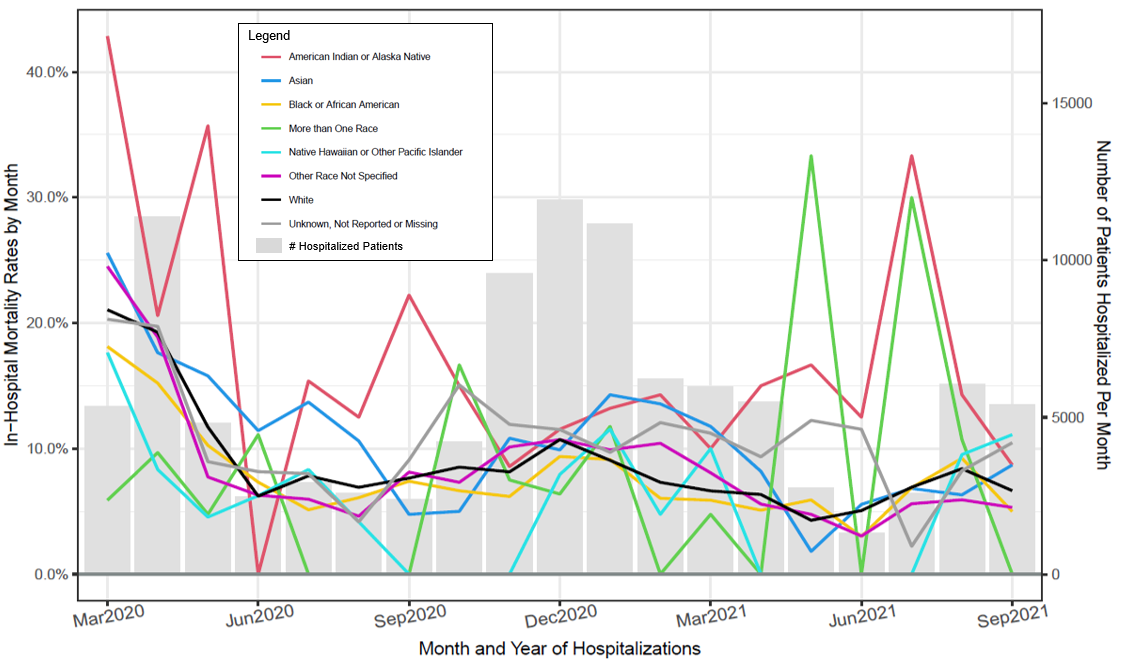


**S8 Fig.** Observed In-Hospital Mortality Rates (Deaths/Hospitalizations) and Number of Patients Hospitalized Per Month with Missing Data for Patient Ethnicity (March 2020 through September 2021).


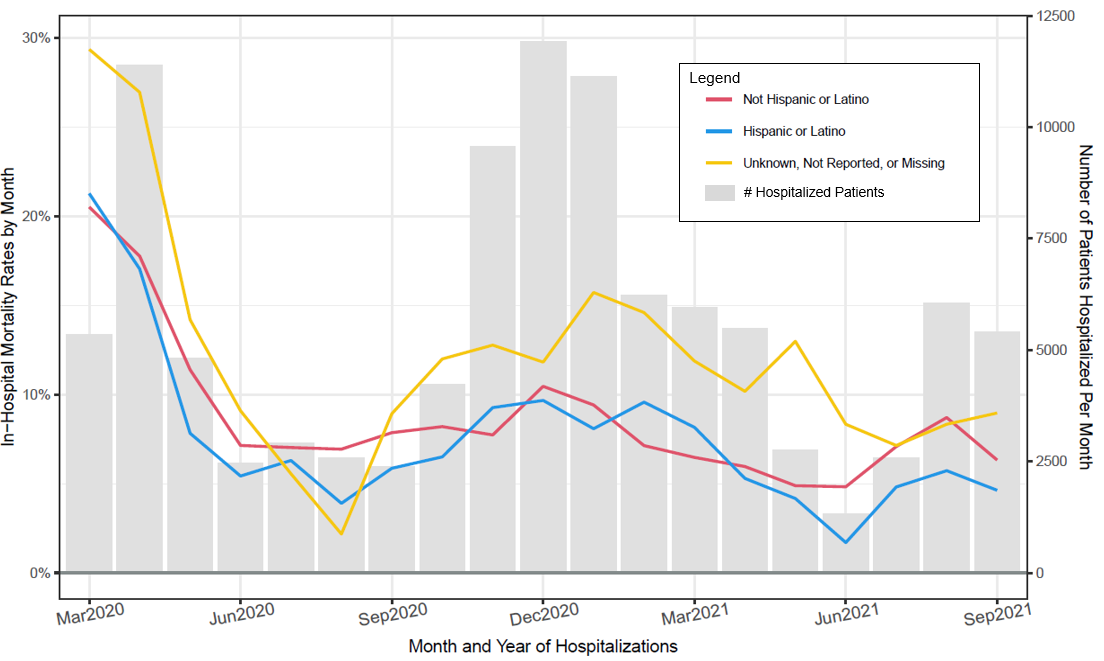


**S9 Fig.** Observed In-Hospital Mortality Rates (Deaths/Hospitalizations) and Number of Patients Hospitalized Per Month with Missing Data for Patient BMI (March 2020 through September 2021).


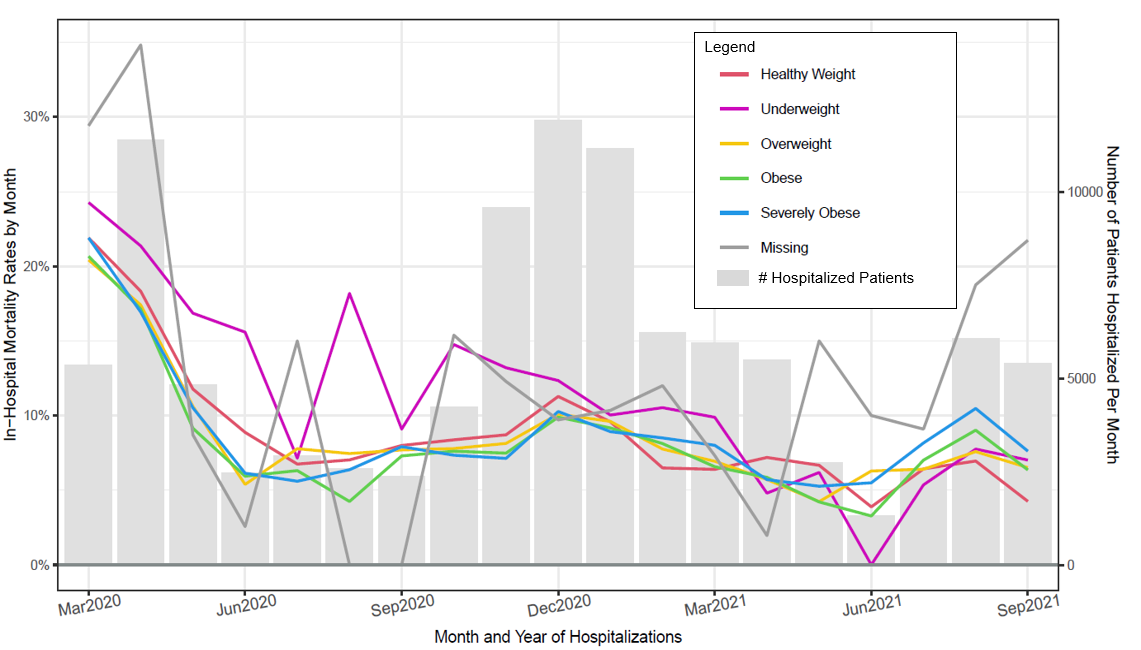


**S10 Fig.** Observed In-Hospital Mortality Rates (Deaths/Hospitalizations) and Number of Patients Hospitalized Per Month with Missing Data for Patient Insurance Status (March 2020 through September 2021).


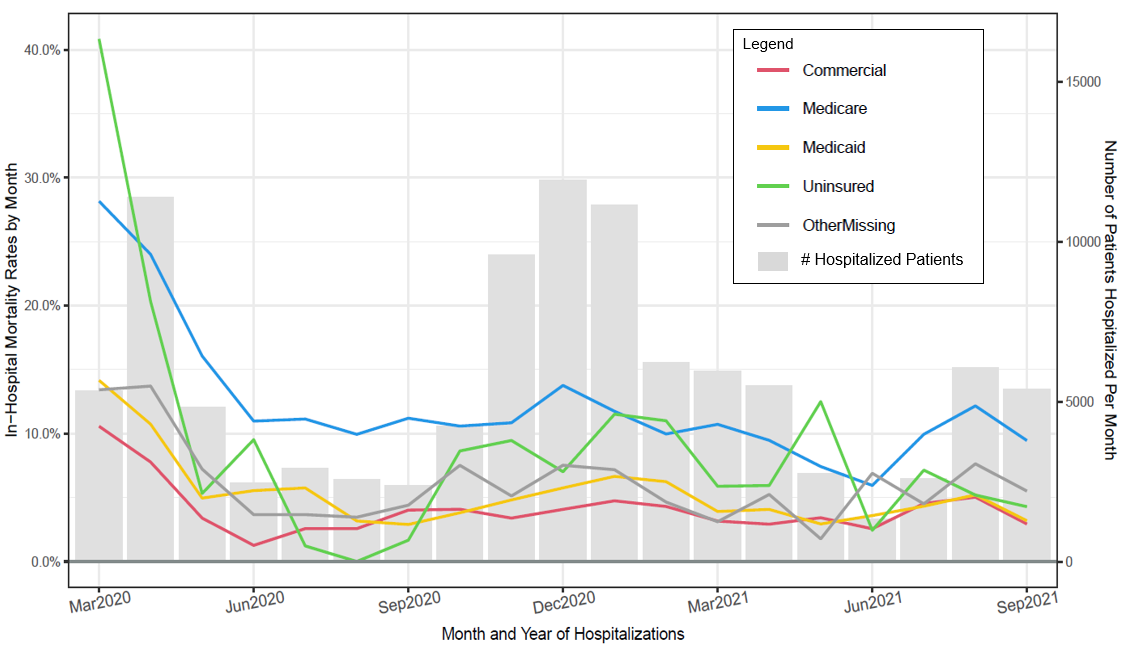


**S11 Fig.** Adjusted In-Hospital Intubation Rates (Intubations/Hospitalizations) and Number of Patients Hospitalized Per Month Across All 21 Health Systems.


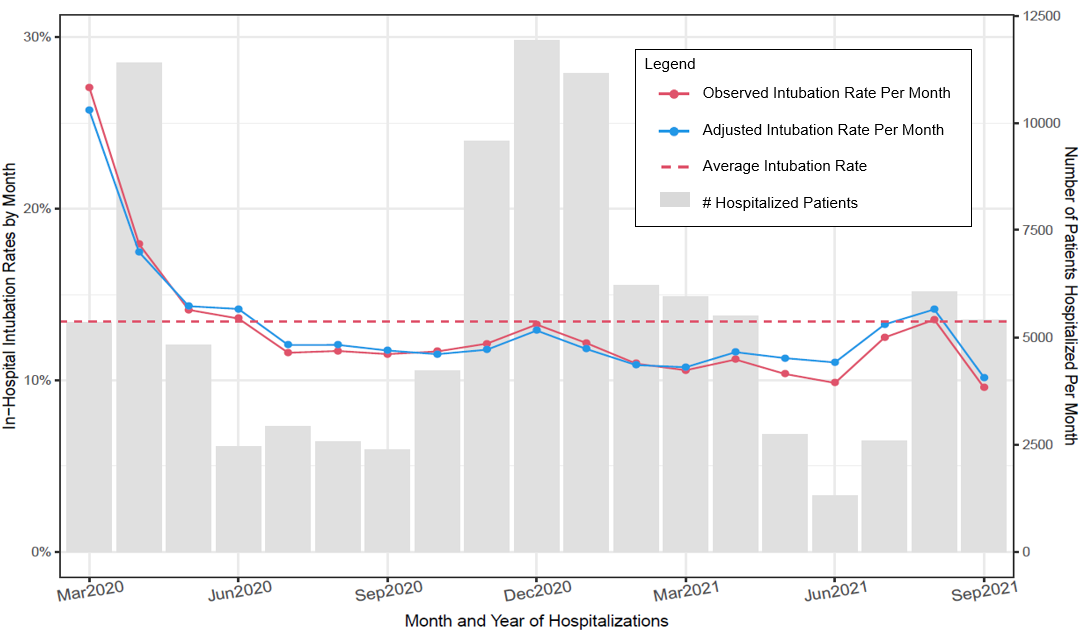


Adjusted for age, sex, race, ethnicity, BMI, insurance status, and Elixhauser Comorbidity Score.

**S12 Fig.** Adjusted In-Hospital ICU Admission Rates (ICU Admissions/Hospitalizations) and Number of Patients Hospitalized Per Month Across All 21 Health Systems.

**
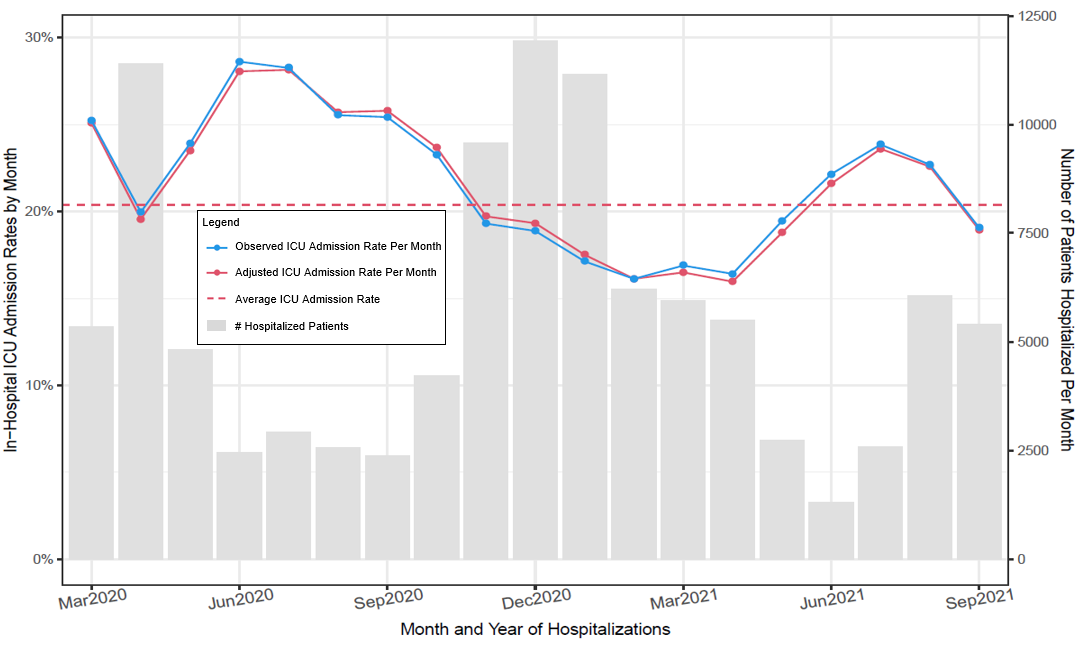
**

Adjusted for age, sex, race, ethnicity, BMI, insurance status, and Elixhauser Comorbidity Score.

**S13 Fig.** Logistic Regression Based Relation of the Elixhauser Comorbidity Score with Mortality, Intubation Status, and ICU Admission Earlier (February-April 2020, Period 1) and Later (July-September 2021, Period 2) in the Pandemic.


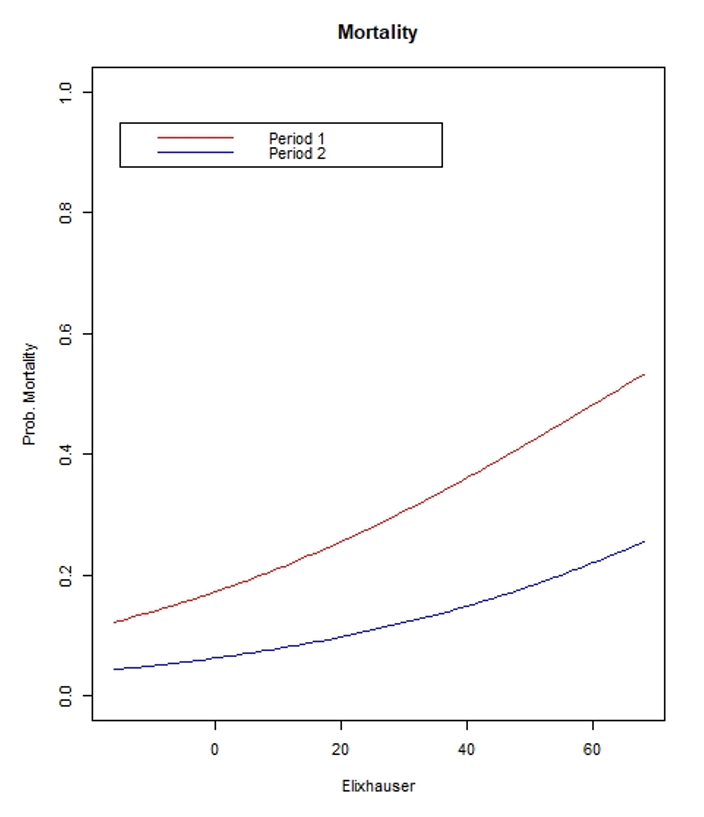


**S14 Fig.** Logistic Regression Based Relation of the Elixhauser Comorbidity Score with Intubation Status Earlier (February-April 2020, Period 1) and Later (July-September 2021, Period 2) in the Pandemic.


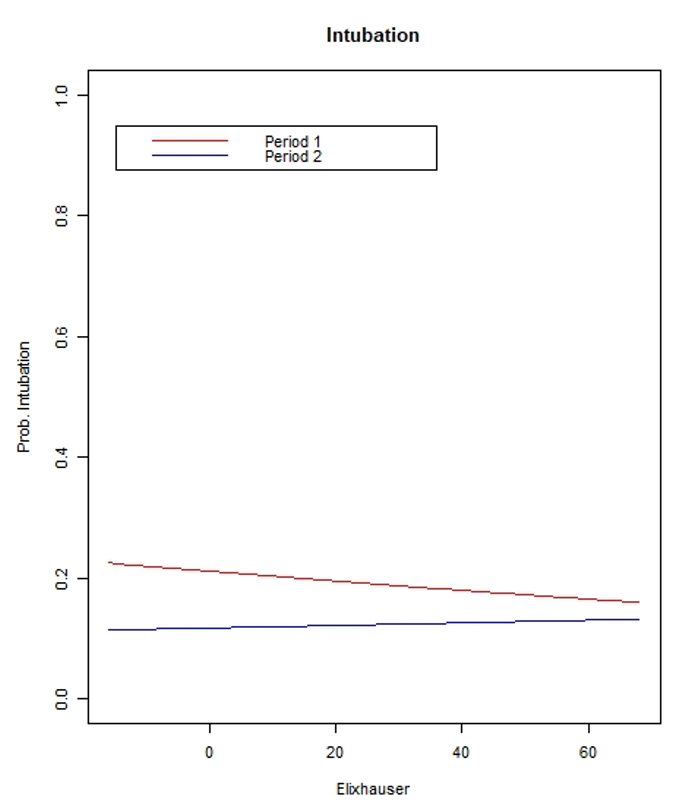


**S15 Fig.** Logistic Regression Based Relation of the Elixhauser Comorbidity Score with ICU Admission Earlier (February-April 2020, Period 1) and Later (July-September 2021, Period 2) in the Pandemic.


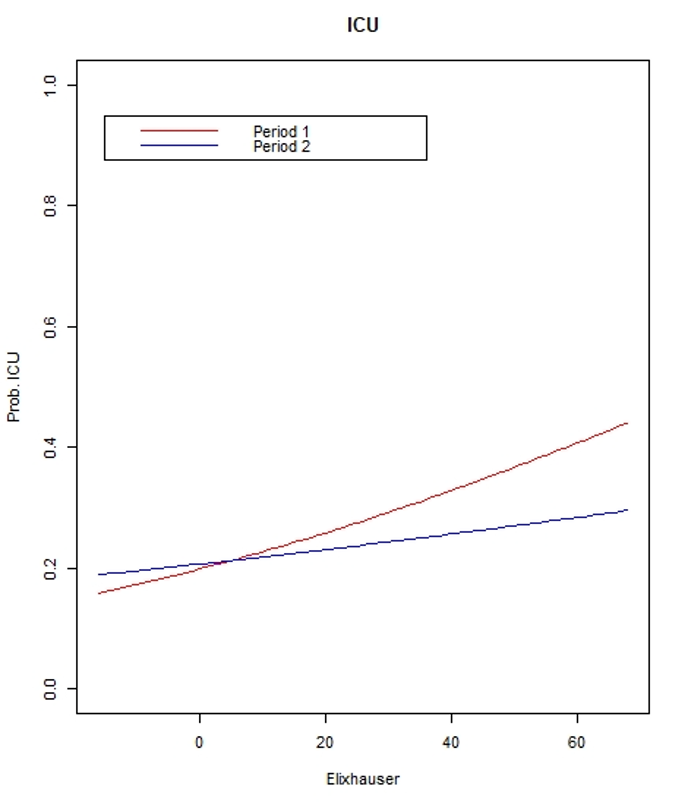


**References**

1. Elixhauser A, Steiner C, Harris DR, Coffey RM. Comorbidity measures for use with administrative data. Med Care. 1998;36(1):8-27. doi:10.1097/00005650-199801000-00004
2. van Walraven C, Austin PC, Jennings A, Quan H, Forster AJ. A modification of the Elixhauser comorbidity measures into a point system for hospital death using administrative data. Med Care. Jun 2009;47(6):626-33. doi:10.1097/MLR.0b013e31819432e5
3. Quan H, Sundararajan V, Halfon P, et al. Coding algorithms for defining comorbidities in ICD-9-CM and ICD-10 administrative data. Med Care. 2005;43(11):1130-1139. doi:10.1097/01.mlr.0000182534.19832.83
4. Asch DA, Sheils NE, Islam MN, et al. Variation in US hospital mortality rates for patients admitted with COVID-19 during the first 6 months of the pandemic. JAMA Intern Med. Apr 1 2021;181(4):471-478. doi:10.1001/jamainternmed.2020.8193
5. Lera, Kay. CEC-UW Participating Health Systems. Retrieved on April 1, 2022 from https://ctri.wisc.edu/cec-uw/covid-map-of-grantee-sites/
